# Dendritic Oligoglycerol Regioisomer Mixtures and Their Utility for Membrane Protein Research

**DOI:** 10.1002/chem.202003991

**Published:** 2020-12-29

**Authors:** Leonhard H. Urner, Katharina Goltsche, Marleen Selent, Idlir Liko, Marc‐Philip Schweder, Carol V. Robinson, Kevin Pagel, Rainer Haag

**Affiliations:** ^1^ Institute of Chemistry and Biochemistry Freie Universität Berlin Takustraße 3 14195 Berlin Germany; ^2^ Physical and Theoretical Chemistry Laboratory University of Oxford South Parks Road Oxford OX1 3QZ UK

**Keywords:** dendrons, detergents, membrane protein purification, oligoglycerol, regioisomers

## Abstract

Dendrons are an important class of macromolecules that can be used for a broad range of applications. Recent studies have indicated that mixtures of oligoglycerol detergent (OGD) regioisomers are superior to individual regioisomers for protein extraction. The origin of this phenomenon remains puzzling. Here we discuss the synthesis and characterization of dendritic oligoglycerol regioisomer mixtures and their implementation into detergents. We provide experimental benchmarks to support quality control after synthesis and investigate the unusual utility of OGD regioisomer mixtures for extracting large protein quantities from biological membranes. We anticipate that our findings will enable the development of mixed detergent platforms in the future.

## Graphical abstract

**The secret lies in the mix**: Regioisomer mixtures of dendritic oligoglycerol detergents have recently attracted attention from experimentalist in membrane protein research. The present study outlines the synthesis of detergent regioisomer mixtures and sheds light on their utility for the purification of membrane proteins. The obtained results point to new avenues for the development of lipid‐like detergent mixtures in membrane protein research.

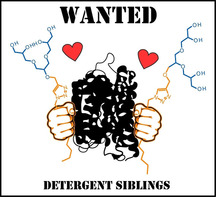

## Introduction

**Scheme 1:**
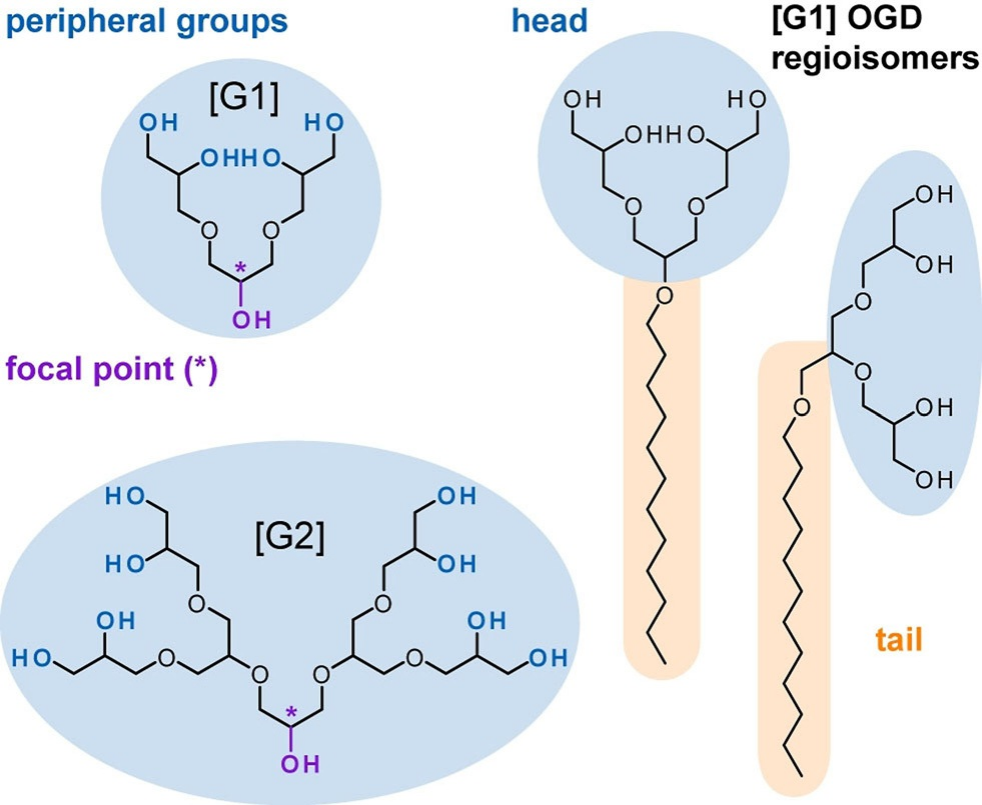
First‐ [G1] and second‐generation [G2] oligoglycerol dendrons contain a single chemically addressable functional group at the core (focal point). The number of hydroxy groups at the periphery and their size vary between the generations. Dendritic OGDs contain a hydrophilic head and a hydrophobic tail. Regioisomers differ in terms of connectivity between glycerol units in the head group. Mixtures of [G1] OGD regioisomers can extract more protein quantities from membranes than individual regioisomers. The origin of this behaviour remains elusive.

Almost 100 years ago, Staudinger pioneered the theory of polymeric structures, which led to the emergence of a research field that we know as macromolecular chemistry.^[1]^ Today, different classes of synthetic macromolecules have been developed, such as linear polymers, hyperbranched polymers, dendrons, and hybrid structures.^[2]^ Unlike synthetic polymers, which are polydisperse, the structure of a dendron is perfectly defined. Dendrons contain a single, chemically addressable functional group at their centre and multiple functional groups at their periphery (Scheme 1). They are classified by generations, which define their overall size and number of peripheral groups. Their monodispersity and the ability to gradually tune their molecular properties are valuable perquisites for structure–property studies.^[3]^

The utility of glycerol for the production of dendrons and related materials has been intensively investigated over the past 20 years.[^2^, ^4^] Glycerol is a green starting material. It is mainly obtained as a by‐product from the vegetable oil industry, while only small amounts are obtained from fossil chemicals.^[5]^ Dendritic oligoglycerol is nonionic, water‐soluble, biocompatible, scalable in size, and straightforward to synthesize. The sum of these advantageous properties makes dendritic oligoglycerol a valuable starting material for structure–property studies. Dendritic oligoglycerol has been used for the production of dendronized polymers,^[6]^ unimolecular micelles,^[7]^ and dendritic OGDs.^[2]^ The first dendritic OGDs were reported by Wyszogrodzka and co‐workers in 2008.^[8]^ Research has focused on understanding the relationship between molecular structure and self‐assembly as well as the use of their aggregates for stabilizing water‐in‐fluorinated oil emulsions^[9]^ and solubilizing drugs or carbon nanotubes.^[2]^

Recently, dendritic OGDs have been identified as versatile tools for the structural analysis of important drug targets: membrane proteins.^[10]^ The modular architecture of dendritic OGDs can be optimized for membrane protein purification, delipidation, and individual applications in native mass spectrometry of proteins.[^10^, ^11^] Interestingly, [G1] OGD regioisomer mixtures can extract larger protein quantities from membranes than individual isomers.[^10a^, ^10c^] So far, [G1] OGD mixtures have been successfully applied for the purification of inner and outer membrane proteins of *Escherichia coli* (*E. coli*) and a functional neurotensin receptor 1 (NTSR1)—a member of the G protein‐coupled receptor family, which is currently one of the most interesting protein classes in pharmacology.^[12]^ However, the synthesis and molecular properties of [G1] OGD regioisomer mixtures have not yet been explored in full detail. Here, we address this shortcoming by investigating i) the general synthesis of OGD regioisomer mixtures and ii) the molecular properties of individual [G1] OGD regioisomers to better understand the utility of their mixtures for extracting large protein quantities.

## Results and Discussion

**Figure 1:**
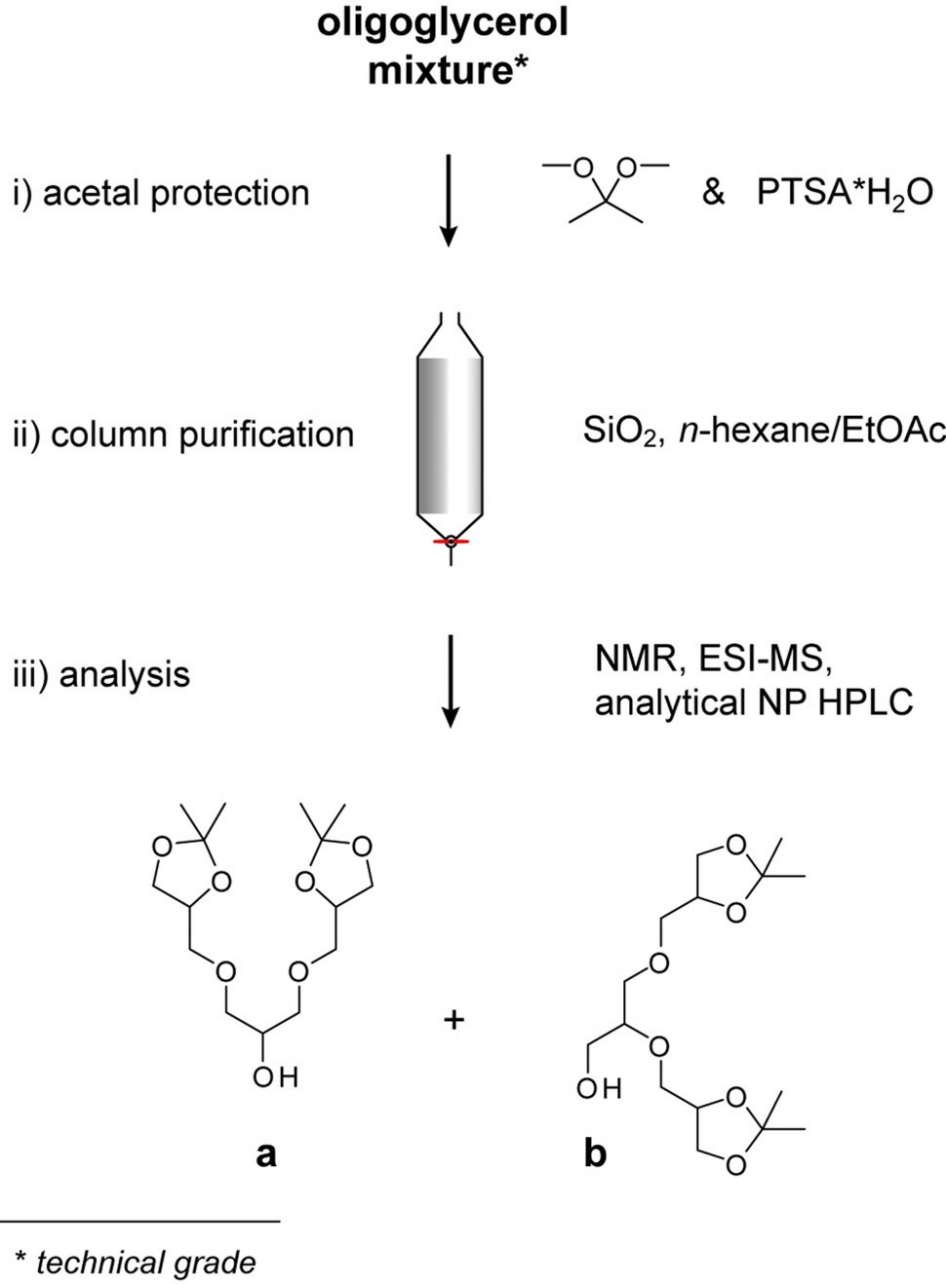
Use of oligoglycerol mixtures for the synthesis of [pG1]‐OH. Two regioisomers of [pG1]‐OH (**a** and **b**) can be co‐purified during acetal protection of oligoglycerol mixtures and column purification.

Acetal‐protected, first‐generation triglycerol [pG1]‐OH is the starting material for dendritic OGDs. It is synthesized by acetal protection of oligoglycerol mixtures (Figure 1).^[13]^ Oligoglycerol mixtures are heterogeneous. They contain different glycerol oligomers and each oligomer population is divided into several regioisomer populations. For triglycerol, seven regioisomers exists and their relative abundances vary with the production method (Figure S1 in the Supporting Information).^[14]^ The exact composition of oligoglycerol mixtures therefore remains a black box. However, they serve as a valuable resource for the synthesis of dendritic OGDs, as it requires only one step to convert them into [pG1]‐OH.^[13]^

Given the heterogeneity of oligoglycerol mixtures, we investigated if triglycerol regioisomers are co‐purified during acetal protection (Figure 1). We screened oligoglycerol from three individual batches and analysed the products by analytical HPLC, electrospray ionization mass spectrometry (ESI‐MS), and NMR spectroscopy. Irrespective of the oligoglycerol batch, we identified two products by HPLC (Figure S2, Table S1). The exact masses of both products obtained from ESI‐MS agreed with those calculated from the sum formula of [pG1]‐OH. Their NMR data, however, showed clear differences (Figure S3). This indicates that the products are regioisomers, which differ in terms of the connectivity between glycerol units. Taking into account previous reports on relative abundances of triglycerol regioisomers,^[14b]^ the co‐purified regioisomers of [pG1]‐OH are likely to be those shown in Figure 1 a, b. Their absolute identity was confirmed by synthesizing both isomers separately and comparing their NMR data to those obtained from the products isolated by HPLC (Figure S3). The relative abundances of **a** and **b** as well as the overall product yields vary with the oligoglycerol batch (Table S1).

To synthesize the next higher generation [pG2]‐OH, two equivalents of [pG1]‐OH were treated with one equivalent of 3‐chloro‐2‐chloromethyl‐1‐propene (methallyl dichloride) under basic conditions.^[13]^ In order to finally yield [pG2]‐OH, the double bond at the focal point of the intermediate [pG2]‐ene was converted to a hydroxy group by ozonolysis and reduction.^[13]^

**Figure 2:**
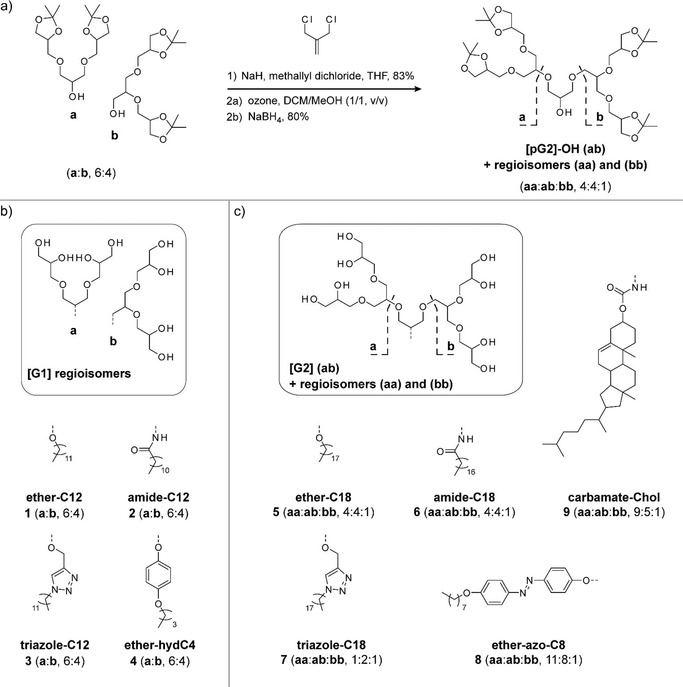
Synthesis of [G1] and [G2] OGD regioisomer mixtures. a) The use of a [pG1]‐OH regioisomer mixture (**a**,**b**) as starting material leads to three [pG2]‐OH regioisomers (**aa**, **ab**, **bb**), which differ in terms of the structure of their triglycerol side chains. Using [pG1]‐OH and [pG2]‐OH regioisomer mixtures as the starting material for detergents leads to b) [G1] and c) [G2] OGD regioisomer mixtures (for further information see the Supporting Information).

To elucidate how using [pG1]‐OH regioisomer mixtures affects the heterogeneity of [pG2]‐OH batches, we performed this reaction on a [pG1]‐OH regioisomer mixture (**a**:**b**, 6:4). For the given reaction conditions, we assumed that i) only one chlorine atom of methallyl dichloride was substituted at a time by **a** or **b** and ii) that the same applied to the formed intermediate (Figure S4). Upon ozonolysis and reduction, we expected three [pG2]‐OH regioisomers that differed in the structure of their triglycerol side chains: **aa**, **ab**, and **bb** (Figures 2 and S4). To prove the formation of a regioisomer mixture, we synthesized **aa** and **bb** separately and compared their HPLC and NMR data to those obtained from the proposed mixture containing **aa**, **ab**, and **bb**. We were not able to distinguish between **aa** and the proposed isomer mixture using normal‐phase HPLC or ^1^H NMR spectroscopy (Figure S5–S6). The two methods might therefore not be suitable to differentiate between regioisomers of [pG2]‐OH. In addition, we analysed the ^13^C NMR spectra between 82 and 66 ppm. This spectral region reflects a structural fingerprint of [pG2]‐OH regioisomers, as it is sensitive to variations in the triglycerol side chains (Figure S6). The signals of **aa** and **bb** were well‐represented in the spectrum of the proposed isomer mixture, which underlines the idea that at least two regioisomers of [pG2]‐OH were formed. Surprisingly, the ^13^C NMR signals of the focal points were sensitive to the structure of the triglycerol side chains (Figure S6). The chemical shifts obtained from **aa** and **bb** differed by less than 1 ppm. Both signals also appeared in the spectrum of the proposed regioisomer mixture. Closer analysis of the relative signal abundances revealed a regioisomer stoichiometry of about 4:4:1 (**aa**:**ab**:**bb**, Table S2).

Both [pG1]‐OH and [pG2]‐OH are starting materials for dendritic OGD architectures.[^10a^, ^13^, ^15^] This motivated us to investigate whether regioisomer ratios would change during detergent synthesis. We applied standard protocols to synthesize the [G1] OGD regioisomer mixtures **1**–**4**, including direct alkylation (**1**); mesylation followed by azidation, reductive amination, and alkylation by amide coupling (**2**); propargylation and alkylation by copper‐catalysed click reaction (**3**); and mesylation followed by alkylation (**4**).[^10a^, ^10b^, ^13^] Irrespective of the synthetic strategy employed, regioisomer ratios of [G1] OGDs were similar to those obtained in the starting material (**a**:**b**, 6:4, Figure 2, Table S2). We therefore conclude that regioisomer ratios of [pG1]‐OH can be fully retained under the experimental conditions employed. We obtained similar results for the [G2] OGD batches **5**–**6** (Table S2). However, we observed a significant shift in regioisomer ratios for the [G2] OGD batches **7**, **8** and **9**; this indicates that the ability to retain regioisomer ratios of [pG2]‐OH varies with the applied synthesis strategy (Figure 2, Table S2).

Having established the synthesis and characterization of OGD regioisomer mixtures, we investigated why [G1] OGD regioisomers can extract more protein quantities from biological membranes than individual regioisomers.^[10a]^ Membranes exhibit a hydrophobic core and a hydrophilic surface, which are formed by a bilayer of lipid molecules. Protein‐containing membranes are poorly soluble in water and exhibit amphiphilic properties.

Following the motto *similia similibus solvuntur* (similar substances will dissolve similar substances), detergents can break up membranes and solubilize both lipids and proteins by forming water‐soluble aggregates. The mechanisms with which detergents extract membrane proteins from membranes has been studied extensively.^[16]^ However, the origin of the unusual utility of [G1] OGD regioisomers for extracting large protein quantities from membranes remains puzzling.

**Figure 3:**
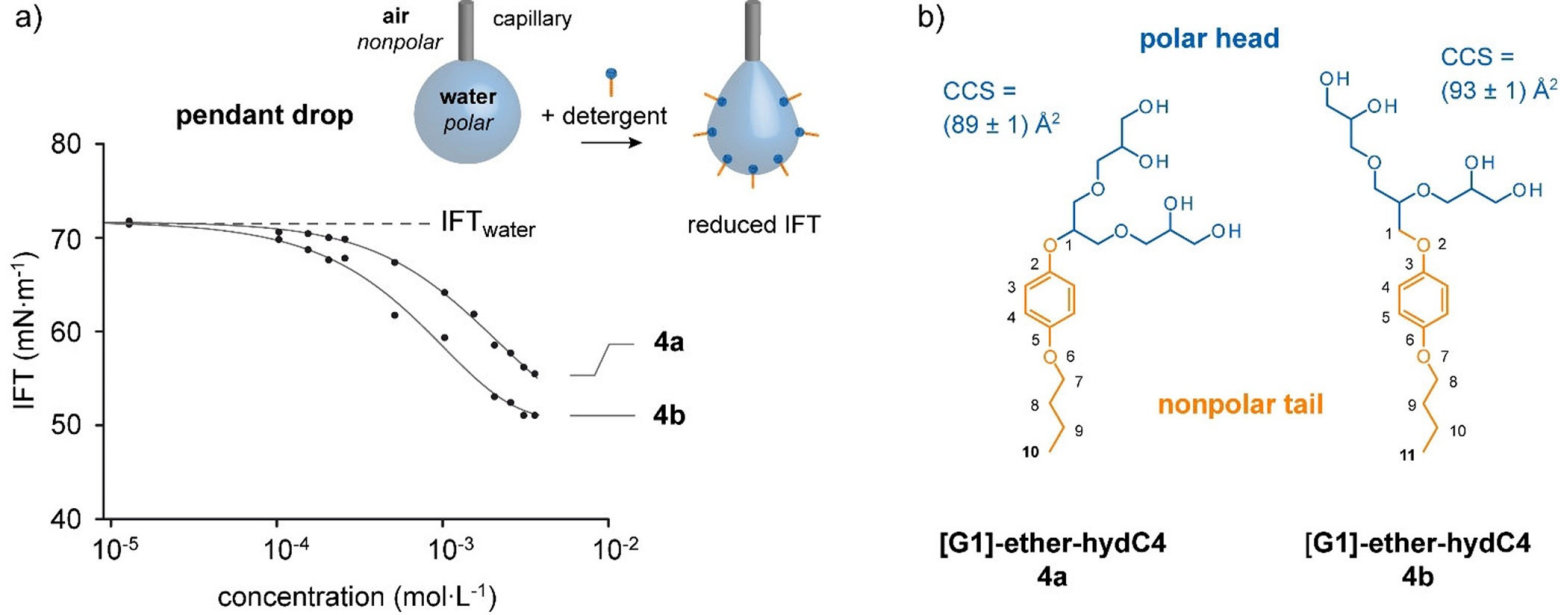
The influence of individual [G1] OGD regioisomers on the interfacial surface tension (IFT) of a water droplet. a) Lower IFT values are obtained from regioisomer **4 b**. b) Changing the focal point in **4 b** increases the length of the nonpolar tail by one methylene unit and increases the size of the polar head group as indicated by a larger collisional cross section (CCS). The [G1] regioisomer **4 b** is more hydrophobic than **4 a** although it has a slightly larger polar head group. CCS values were calculated from model structures using the projection approximation algorithm (for further information see the Supporting Information).

The utility of a detergent for protein extraction depends on its molecular structure.[^10a^, ^17^] This motivated us to study the molecular properties of individual [G1] OGD regioisomers in more detail using the pendant drop method. The air surrounding a water droplet is hydrophobic and water is hydrophilic. The lateral polarity distribution of the air–water interface is similar to that of a membrane. Detergents adsorb at the air–water interface and reduce the interfacial surface tension (IFT).^[18]^ Lower IFT values were obtained for **4 b**, which indicates that its structure is more hydrophobic than **4 a** (Figure 3).^[19]^ This agrees with the observation that the change of the focal point in **4 b** increases the length of the hydrophobic tail effectively by the length of one methylene unit. At the same time, collision cross section (CCS) values calculated from model structures suggest that the difference in focal point structure also increases the size of the head of **4 b** (Figure 3). Increasing the size of the hydrophilic head group usually decreases the hydrophobicity of a dendritic OGD.^[10a]^ However, the opposite seems to be the case for [G1] OGD regioisomers.

An alternative measure for the hydrophobicity of organic compounds is the partition coefficient (P), which is defined as a ratio of concentrations of a solute between two solvents. Given that one solvent is water and the other a non‐polar solvent, the logarithm of *P* (log *P*) becomes a measure of hydrophobicity. Conversely, the log *P* values predicted by ChemDraw for both isomers are similar (e.g., log *P* of **1 a** and **1** ***b*** **=**2.23). This led to the question whether the differences in hydrophobicity indicated by surface tension experiments are relevant for solution properties of [G1] OGDs.

To address this question, we first compared the isocratic elution profiles of [G1] OGD regioisomer mixtures from reversed‐phase HPLC column material. The retention times of the isomers **1 a**, **2 a**, and **3 a** were consistently shorter, thus pointing to a less efficient interaction with the hydrophobic stationary phase (Figure S7). This underlines that symmetric [G1] OGD regioisomers **1 a**, **2 a**, and **3 a** indeed exhibit a less hydrophobic character, irrespective of the linker between head and tail. Second, we investigated if the change in hydrophobicity is affecting the aggregation behaviour. For this purpose, we determined the *critical aggregation concentration* (*cac*) of **3**, **3 a**, and **3 b** by means of dynamic light scattering.^[20]^ The *cac* of **3 a** (470 μm) is higher than that of **3 b** (390 μm), which agrees with its reduced hydrophobicity. Moreover, the *cac* of the mixture **3** (550 μm) is higher than the *cac* values of the individual isomers **3 a** and **3 b**. A similar trend was observed among the regioisomer mixture **1** (700 μm) and the individual isomer **1 a** (490 μm). This indicates that increasing the heterogeneity in [G1] OGD detergent mixtures lowers their tendency to form detergent aggregates.

**Figure 4:**
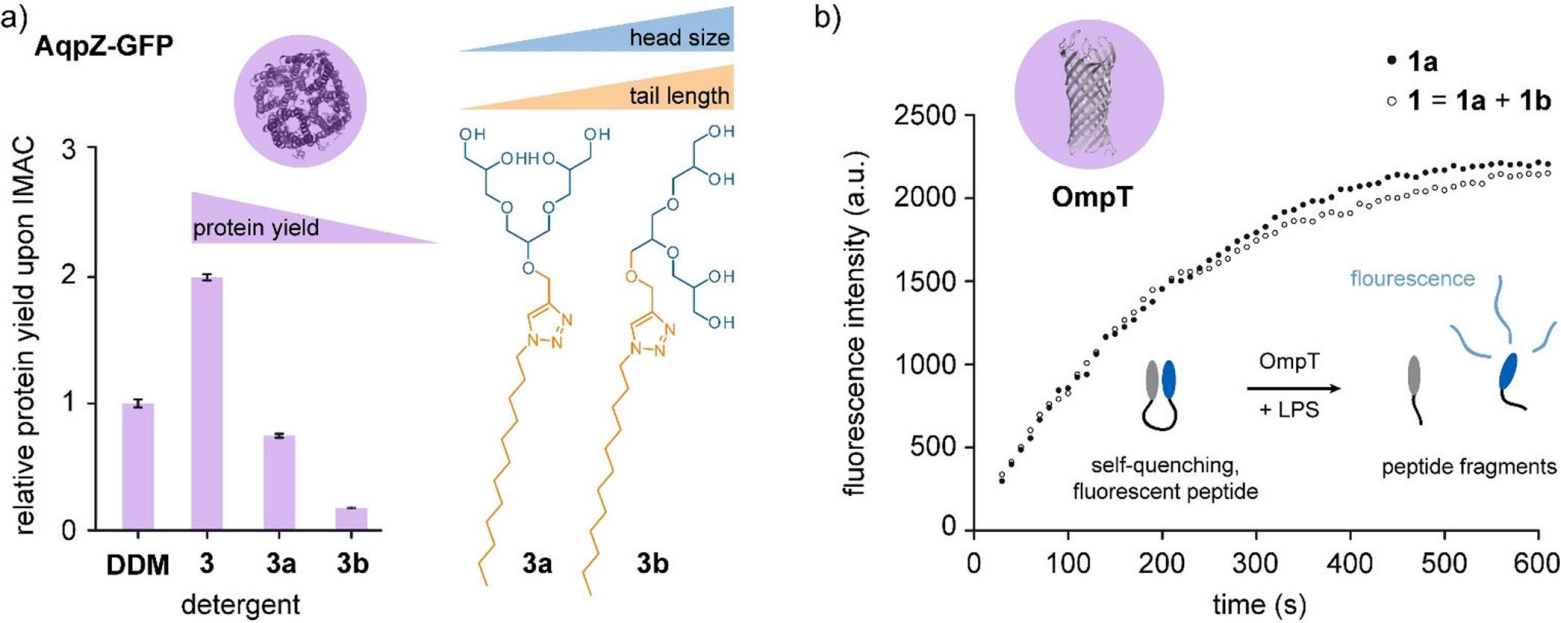
Use of [G1] OGDs for purifying membrane proteins. a) Increasing the size of the head and length of the tail among **3 a** and **3 b** reduces achievable protein yields. Higher protein yields were obtained from the [G1] OGD regioisomer mixture **3**. b) The proteolytic activity of OmpT against a self‐quenching, fluorescent peptide is not strongly affected by the presence or absence of [G1] OGD regioisomer **1 b**. Mixing detergents with slightly different hydrophobic tails and head groups might not affect protein activity but provides a better mimic for heterogeneous lipid membranes during extraction.

To study the relevance of the outlined property differences for membrane protein purification, we re‐investigated the utility of [G1] OGD regioisomers **3 a** and **3 b** to extract the aquaporin channel (AqpZ) and ammonia channel (AmtB) from native membranes of *E. coli*.^[10a]^ The protein quantities were normalized to those obtained from *n*‐dodecyl‐β‐d‐maltoside (DDM), which is a standard detergent in structural biology.^[21]^ Lower protein quantities were obtained from regioisomer **3 b** (Figures 4 and S8). This agrees with the hypothesis that increasing the length of the tail as well as the size of the head group decreases the ability of OGDs to solubilize biological membranes.^[10a]^ In contrast, higher protein yields were obtained from the [G1] OGD regioisomer mixture **3**. We assume that mixing detergents with slightly different hydrophobic tails and head groups provides a better mimic for heterogeneous lipid membranes than monodisperse detergent batches. Moreover, the reduced propensity of [G1] OGD regioisomer mixtures to form detergent aggregates leads likely to a higher concentration of detergent monomers in solution.^[22]^ This could support protein extraction, because the uptake of detergent monomers into membranes is a potential key step for initializing membrane solubilization.^[23]^

Previous detergent exchange experiments on the outer membrane protein F revealed that [G1] OGD regioisomer mixtures and individual regioisomers can form stable proteomicelles in the absence of membranes.^[10a]^ Considering these results, we finally investigated whether mixing [G1] OGD regioisomers can affect the activity of membrane proteins. The outer membrane protease T (OmpT) was selected as model system because it is compatible to [G1] OGDs.^[10a]^ Briefly, the protein was isolated from inclusion bodies of *E. coli* and refolded using the [G1] OGD regioisomer mixture **1**. Refolded OmpT was incubated with the self‐quenching, fluorescent peptide and lipopolysaccharide (LPS). Binding to LPS is required to increase the proteolytic activity of OmpT.^[24]^ The digestion of the self‐quenching peptide by OmpT produces a fluorescent peptide fragment. Therefore, the activity of OmpT could be monitored by fluorescence spectroscopy in a time‐resolved manner (Figure 4).[^10a^, ^25^] Control experiments with the individual regioisomer **1 a** revealed that the proteolytic activity is not significantly affected by the absence of regioisomer **1 b**.

## Conclusions

In summary, we have investigated the synthesis of oligoglycerol regioisomer mixtures. We found that triglycerol regioisomers are co‐purified during acetal protection of oligoglycerol mixtures. The ability to retain regioisomer ratios during detergent synthesis varies with the dendron generation and synthesis strategy. The NMR raw data provided with this paper will facilitate the quality control of dendritic detergents after synthesis. Both [G1] OGD regioisomers and individual isomers can form stable proteomicelles and retain the activity of membrane proteins in the absence of membranes. However, changing the focal point structure in [G1] OGD regioisomers simultaneously increases the size of the head and overall hydrophobicity of the detergent, thus leading to a more lipid‐like environment in their mixtures. Furthermore, aggregates formed by [G1] OGD regioisomer mixtures may co‐exist with a larger population of detergent monomers in solution compared to aggregates formed by individual isomers. These unusual properties are linked to the utility of detergent mixtures for extracting large protein quantities from membranes. We anticipate that heterogeneous, lipid‐like detergent mixtures will facilitate the extraction and analysis of difficult membrane proteins in the future.

## Experimental Section

Details of the synthesis and experiments are provided in the Supporting Information. NMR raw data can be downloaded from the OSF website (https://doi.org/10.17605/OSF.IO/TJXSR). The synthesis protocols of [G1] OGD batches **1**, **3**, and [G2] OGD batches **5**, **8**, and **9** have been reported previously.[^10a^, ^10b^] The protocols for the synthesis of **2**, **4**, **6**, and **7** have not been published before.

## Conflict of interest

The authors declare no conflict of interests.

## Supporting information

As a service to our authors and readers, this journal provides supporting information supplied by the authors. Such materials are peer reviewed and may be re‐organized for online delivery, but are not copy‐edited or typeset. Technical support issues arising from supporting information (other than missing files) should be addressed to the authors.

Supplementary
