# Supplementary material for: Dendritic Oligoglycerol Regioisomer Mixtures and Their Utility for Membrane Protein Research
Source: Chemistry. 2020 Dec 29;27(7):2537–42. doi: 10.1002/chem.202003991 (PMC7898920; doi:10.1002/chem.202003991)
Supplement: Supplementary file 1 — Supplementary [file CHEM-27-2537-s001.pdf]

Chemistry–A European Journal

Supporting Information

Dendritic Oligoglycerol Regioisomer Mixtures and Their Utility for Membrane Protein Research

Leonhard H. Urner,*[a] Katharina Goltsche,[a] Marleen Selent,[a] Idlir Liko,[b] Marc-Philip Schweder,[a] Carol V. Robinson,[b] Kevin Pagel,[a] and Rainer Haag[a]

Table of Contents

1. Supplementary Figures 2
2. Supplementary Tables 7
3. Supplementary Methods 9
3.1 General Information about Synthesis 9
3.2 HPLC 10
3.3 Synthesis 11
  3.3.1 [pG1]-OH Regioisomer Mixture (a,b) 11
  3.3.1 [pG1]-OH (a) 11
  3.3.2 [pG1]-OH (b) 12
  3.3.3 [pG2]-OH Regioisomer Mixture (aa,ab,bb) 15
  3.3.3 [pG2]-OH (aa) 17
  3.3.4 [pG2]-OH (bb) 18
  3.3.5 [G1] OGD Regioisomer Mixture 2 19
  3.3.6 [G1] OGD Regioisomer Mixture 4 21
  3.3.7 [G2] OGD Regioisomer Mixture 6 24
3.4 Collision Cross Section Calculation 30
3.5 Pendant Drop Method 33
3.6 Estimation of logP Values 33
3.7. Critical Aggregation Concentration 33
3.8 Membrane Protein Purification 34
3.9 Monitoring the Activity of Outer Membrane Protease T 34
4. Literature 35

1. Supplementary Figures

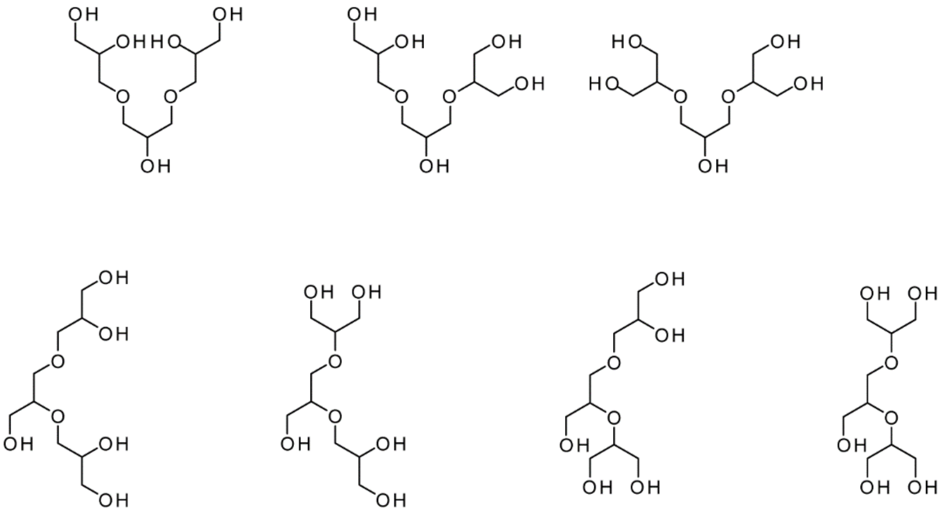

Figure S1. Overview about seven possible regioisomers of triglycerol. The structures shown in this figure have identical sum formulas, but differ in terms of connectivity between glycerol units.

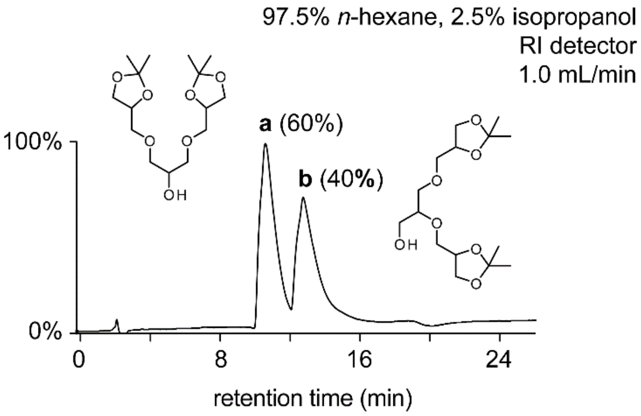

Figure S2. Chromatogram obtained from normal-phase HPLC analysis of [pG1]-OH that was synthesized from oligoglycerol (distributor: Fluka, product code: 17782). The ratio between both products **a** and **b** is about 6:4. Both **a** and **b** are regioiosmers of [pG1]-OH. The composition of the mobile phase (*n*-hexane, isopropanol), detection system, and flow rate (mL/min) are shown. The injection peak is labeled with *Inj.* For further information about the HPLC setup see Supplementary Methods.

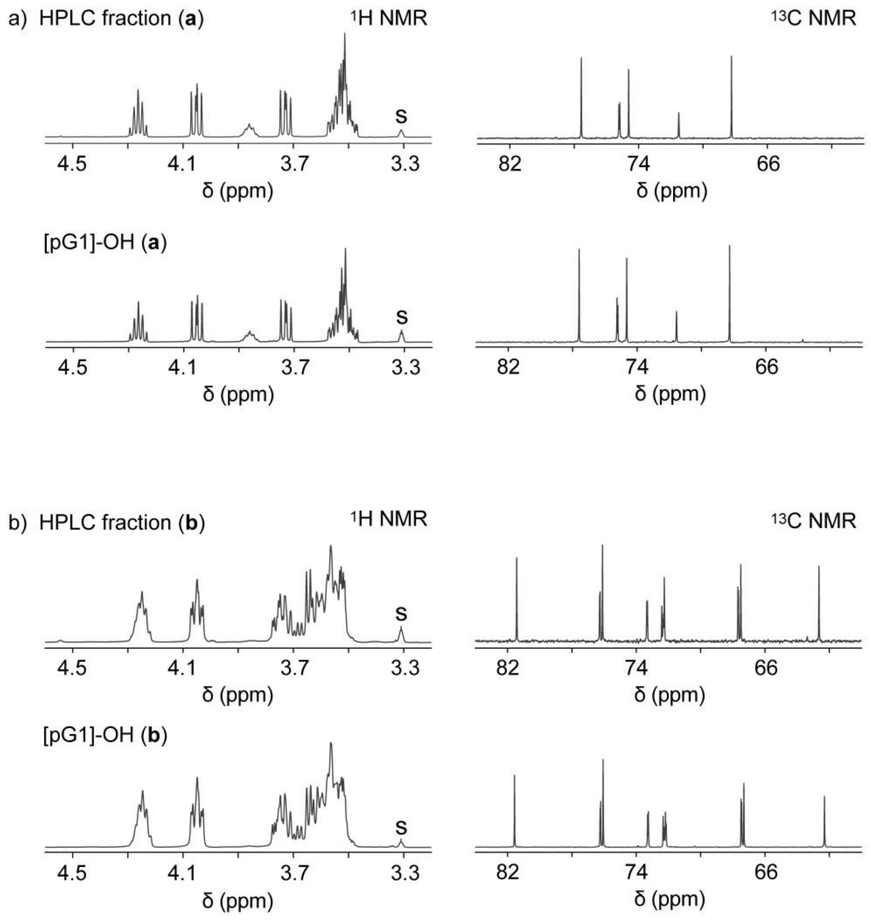

Figure S3. NMR data obtained from a) HPLC fraction **a** and b) HPLC fraction **b** together with the spectra obtained from separately synthesized [pG1]-OH regioisomers **a** and **b**. The spectra confirm that both [pG1]-OH regioisomers are co-purified during acetal protection of oligoglycerol mixtures (distributor: Fluka, product code: 17782). NMR samples were prepared in deuterated methanol (MeOD-d_4).

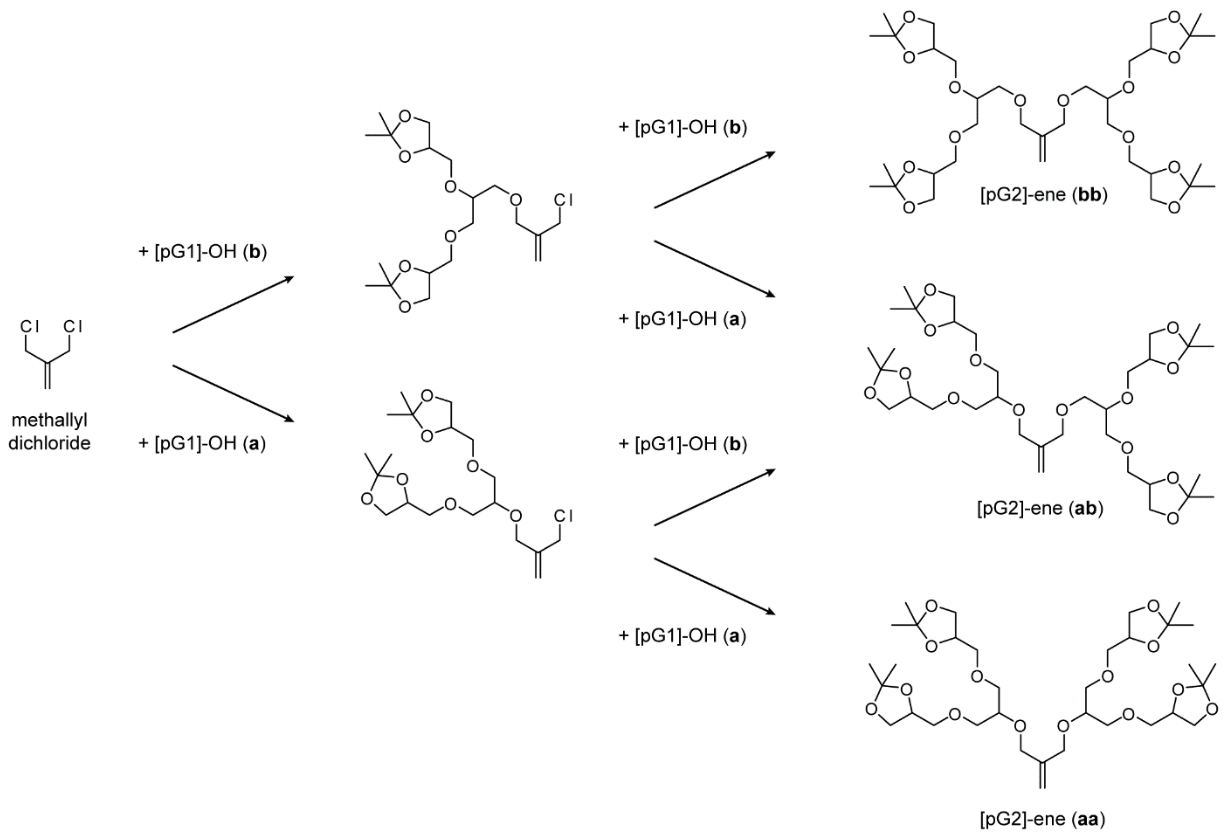

Figure S4. Proposed reaction pathways which could explain the formation of [pG2]-ene regioisomer mixtures. The reaction pathways apply to methallyl dichloride that is reacted with equimolar amounts of [pG1]-OH regioisomers **a** and **b** under the experimental conditions employed. For further information about synthesis see Supplementary Methods.

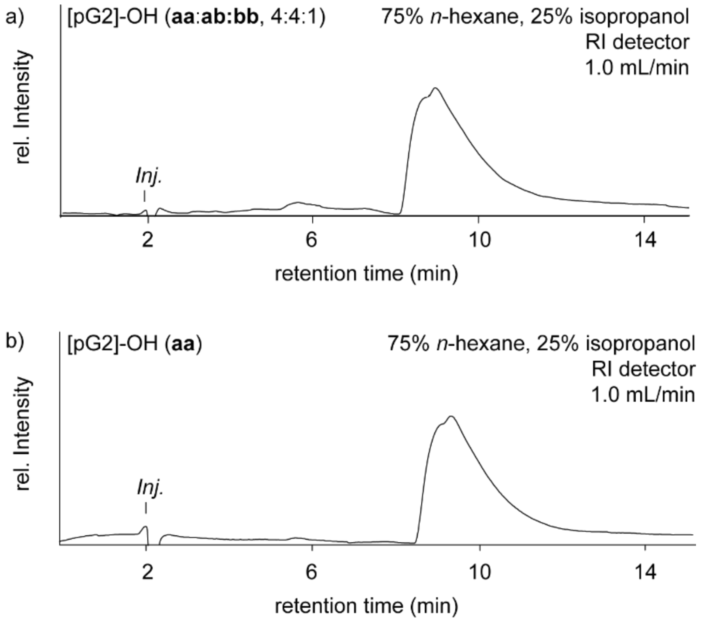

Figure S5. Chromatogram obtained from normal-phase HPLC analysis of a) [pG2]-OH regioisomer mixture (**aa**:**ab**:**bb**, 4:4:1) and b) separately synthesized [pG2]-OH regioisomer **aa**. The composition of the mobile phase (*n*-hexane, isopropanol), detection system, and flow rate (mL/min) are shown. The injection peak is labeled with *Inj.* Regioisomers of [pG2]-OH cannot be separated by normal-phase HPLC under the experimental conditions employed. For further information about the HPLC setup see Supplementary Methods.

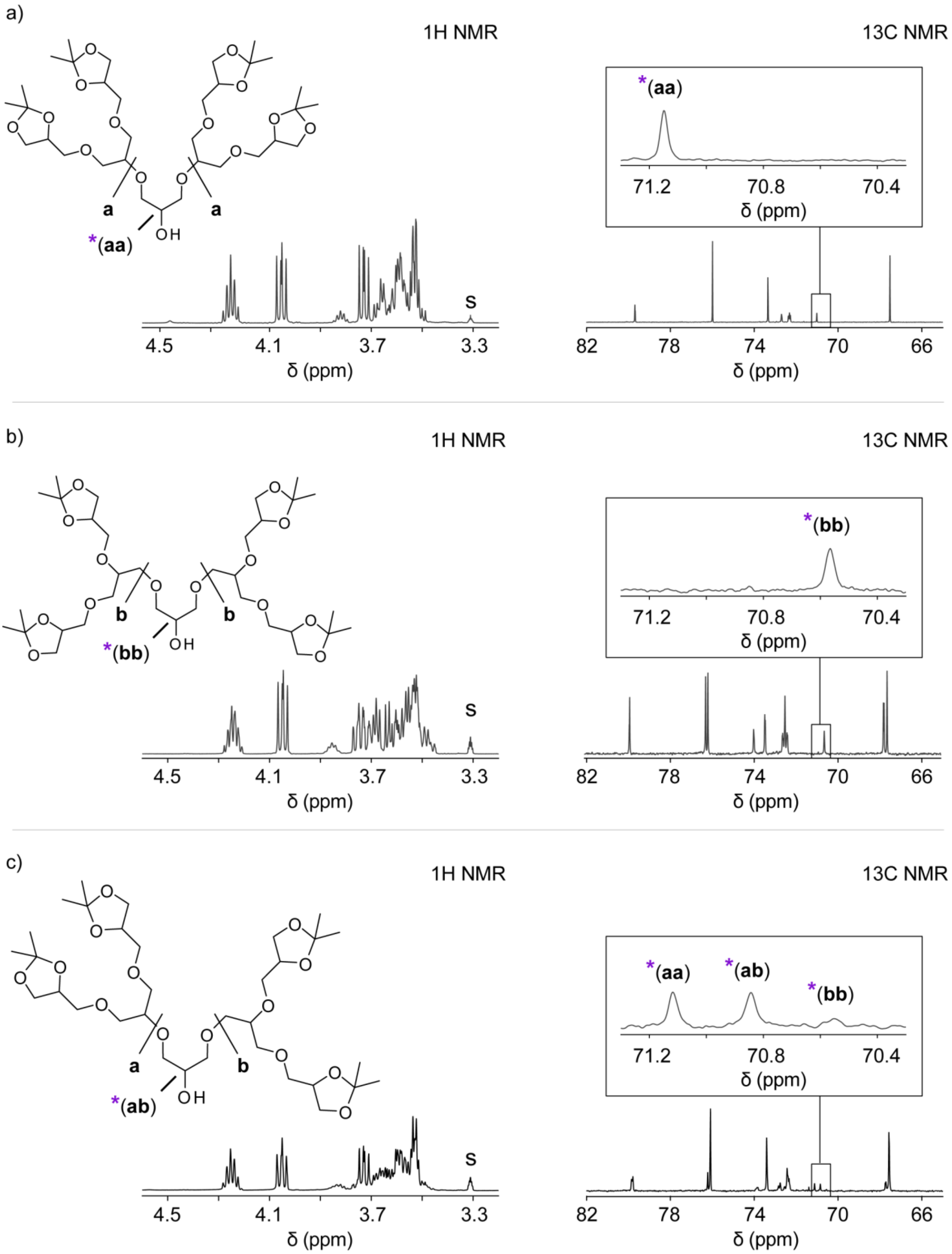

Figure S6. NMR data obtained from a) [pG2]-OH (**aa**), b) [pG2]-OH (**bb**), and c) [pG2]-OH regioisomer mixture (**aa**:**ab**:**bb**, 4:4:1). 13C NMR signals of the focal points provide information about identity and relative abundance of [pG2]-OH regioisomers. Focal point signals are labelled with an asterisk. NMR samples were prepared in deuterated methanol (MeOD-d_4).

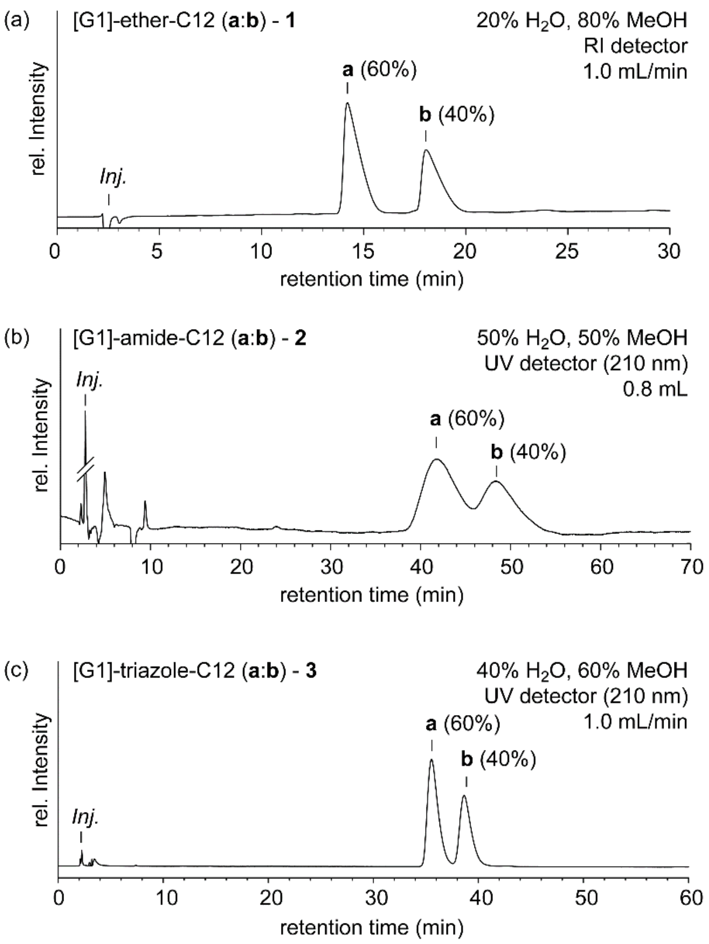

Figure S7. RP-HPLC chromatograms obtained from (a) – (c) [G1] OGD regioisomer mixtures **1**, **2**, and **3**. Shorter retention times were obtained for the isomers **1a**, **2a**, and **3a**, thus indicating that these isomers exhibit a less hydrophobic character. Information about the mobile phase composition (H2O, MeOH), detector system, *e.g.*, UV detector or RI detector, and flow rate (mL/min) are given. The injection peak is labelled with *Inj.*

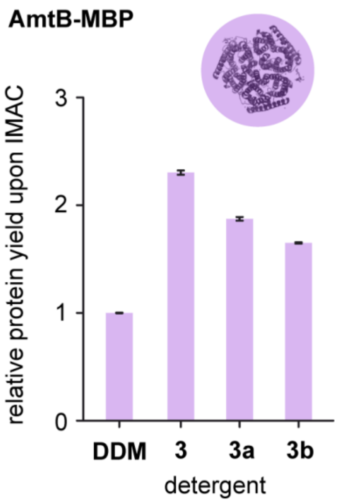

Figure S8. Utility of [G1] OGDs for the purification of the membrane protein AmtB-MBP. Increasing the size of the head and length of the tail among **3a** and **3b** reduces the obtainable protein yields. Higher yields of AmtB-MBP were obtained from the [G1] OGD regioisomer mixture **3**.

2. Supplementary Tables

Table S1. Information about oligoglycerol mixtures, including vendors, product codes, ratios between both [pG1]-OH regioisomers (**a**:**b**) obtained upon acetal protection, and overall yields. Ratios between [pG1]-OH regioisomers and overall yields vary with from batch to batch.

**vendor**	**product code**	**a:b**[a]	**yield (%)**
Fluka	17782	6:4	60
Solvay	TM4516	24:1	80
Sigma-Aldrich	17782 Aldrich	99:1	< 10

[a] determined by analytical normal-phase HPLC.

Table S2. Overview about regioisomer ratios obtained in different products, which were synthesized from Fluka oligoglycerol. Regioisomer ratios of [pG1]-OH, [pG2]-OH, related [G1] (**a**:**b**) as well as [G2] (**aa**:**ab**:**bb**) detergent batches are shown. Chemical shifts of focal points obtained by 13C NMR analysis of [pG2]-OH and [G2] OGD regioisomer mixtures are also shown. Regioisomer ratios of [pG1]-OH can be retained over multiple synthesis steps, while [pG2]-OH regioisomer ratios can vary.

**entry**	**a**:**b**[a]	**aa**:**ab**:**bb**[b]	**chemical shifts (ppm)**[b]
[pG1]-OH	6:4	-	-
**1** [G1]-ether-C12	6:4	-	-
**2** [G1]-amide-C12	6:4	-	-
**3** [G1]-triazole-C12	6:4	-	-
**4** [G1]-ether-hydC4	6:4	-	-
[pG2]-OH	-	4:4:1	71.5 – 70.4
**5** [G2]-ether-C18	-	4:4:1	79.6 – 78.9
**6** [G2]-amide-C12	-	4:4:1	50.8 – 49.9
**7** [G2]-triazole-C18	-	1:2:1	79.2 – 78.4
**8** [G2]-ether-azoC8		11:8:1	78.3 – 77.7
**9** [G2]-carbamate-Chol		9:5:1	52.5 – 51.7

[a] determined by analytical normal-phase HPLC. [b] determined by 13C NMR (inverse-gated) in MeOD-d_4.

Table S3. Summary of results obtained from pendant drop experiments including detergent concentrations (c), interfacial surface tension (IFT) average values (n = 3), and standard deviation (±SD).

[G1]-ether-hydrC4 (4a)			[G1]-ether-hydrC4 (4b)		
c(mol·L^{-1})	IFT(mN·m^{-1})[a]	±SD(mN·m^{-1})	c(mol·L^{-1})	IFT(mN·m^{-1})[a]	±SD(mN·m^{-1})
1.28714E-5	72	0.035	1.28714E-5	71.4	0.068
1.02972E-4	70.5	0.018	5.14858E-5	70.5	0.011
1.54457E-4	70.2	0.039	1.02972E-4	69.8	0.050
2.05943E-4	69.9	0.020	1.54457E-4	68.7	0.18
2.57429E-4	69.8	0.0064	2.05943E-4	67.6	0.085
5.14858E-4	67.4	0.039	2.57429E-4	67.8	0.058
0.00103	64.5	0.054	5.14858E-4	61.7	0.30
0.00154	61.9	0.12	0.00103	59.3	0.14
0.00206	58.4	0.028	0.00206	53.0	0,13
0.00257	57.7	0.028	0.00257	52.4	0.13
0.00309	55.4	0.15	0.00309	51.1	0.095
0.0036	56.0	0.087	0.0036	51.1	0.22
0.00412	55.4	0.020	0.00412	48.4	0.25

[a] determined by means of the pendant drop method

Table S4. Summary of detergents, [G1] OGD regioisomer ratios (a:b), relative protein quantities [P], and standard deviations (±SD) obtained from the extraction of AqpZ-GFP and AmtB-MBP from native *E. coli* membranes. As described above, the protein quantities were obtained from a previously published paper.[1]

Protein	Detergent	a:b	[P][a]	±SD
AqpZ-GFP	DDM	-	1	0.012
	**3** (= **3a** + **3b**)	6:4	1.99	0.010
	**3a**	1:0	0.74	0.0063
	**3b**	0:1	0.17	0.0010
AmtB-MBP	DDM	-	0.35	0.0012
	**3** (= **3a** + **3b**)	6:4	2.3	0.0085
	**3a**	1:0	1.87	0.0073
	**3b**	0:1	1.65	0.0027

[a] protein quantities were determined by UV/VIS spectroscopy (A280nm for AmtB-MBP, A485nm for AqpZ-GFP) and normalized to the values obtained from DDM[1]

3. Supplementary Methods

3.1 General Information about Synthesis

Synthesis of [pG1]-OH and [pG2]-OH regioisomers were conducted in analogy to previously published procedures.[2] The acetal protection of oligoglycerol mixtures was done as described previously.[2a] The syntheses of regioisomer mixtures and individual regioisomers have been subjects of a previous invention.[3] Synthesis protocols that led to the obtainment of [G1] OGD batches **1** and **3** as well as [G2] OGD batches **5**, **8**, and **9** have been published before.[1, 4] Synthesis protocols which led to the obtainment of **2**, **4**, **6**, and **7** have not been published before in journal format. The starting materials, lab equipment, and work-flows used to obtain the here-described compounds were similar to those from previously published protocols:[1, 4-5]

Chemicals were purchased from Sigma-Aldrich (Germany), Acros Organics (Germany), Alfa Aesar (Germany), Fluka (Germany), Fisher Scientific (Germany), Merck (Germany), TCI (Germany). Chemicals were used as supplied. Ethyl acetate (EtOAc), *n*-hexane, and *n*-pentane were distilled before they were used. Other solvents, such as methanol (MeOH), dimethylformamide (DMF), *tert*-butanole (^{t}BuOH), and dichloromethane (DCM) were used as supplied. Dry solvents were purchased in bottles sealed with a septum or tapped from a solvent purification system (MS-SPS-800) that was purchased from M. Braun (Germany). Deionized water used for synthesis was provided by a deionization system installed in the Freie Universität's Institute of Chemistry and Biochemistry. Argon was purchased from Linde (Germany). For working under dry and oxygen-free reaction conditions, chemicals and solvents were handled under argon atmosphere. To support dry conditions the glassware was evacuated, heated up to 300 °C with a heat gun, and filled with argon.

Monitoring of reactions and purification procedures was achieved by normal-phase thin-layer chromatography (TLC) analysis. TLC plates (DC-Fertigfolien ALUGRAM® Xtra SIL G/UV254) based on silica (SiO_2) were purchased from Macherey-Nagel (Germany). Silica gel (60 M) for preparative normal-phase column chromatography was purchased from Macherey-Nagel. For normal-phase TLC analysis and manual column purification mixtures of organic solvents (*v:v*) were prepared. If necessary, MeOH was added in percent per volume to the prepared mixtures (*v*:*v* + *v*%). TLC plates were either analysed under UV irradiation (254 nm) using a lamp from CAMAG (Germany) or by staining the TLC plates with cerium reagent (940 mL H_2O, 60 mL H_2SO_4, 25 g molybdic acid, 10 g cerium(IV) sulfate). For the staining process, the TLC plates were fully submerged into the cerium reagent, excess of staining reagent was wiped off with cellulose, and the plate was heated up to 300 °C with a heat gun until staining was completed.

Mass spectra were acquired on an Agilent 6210 ESI-TOF (ESI-ToF) from Agilent Technologies (Santa Clara, CA, USA). The solvent flow rate was adjusted to 4 μL/min and the spray voltage was set to 4 kV. Drying gas flow rate was set to 15 psi (1 bar). All other parameters were adjusted for a maximum

abundance of the relative $[M+H]^+$. The instrument was operated by the Core Facility BioSupraMol of the Freie Universität Berlin.

1H NMR, 13C NMR and DEPT135 spectra were acquired using the following NMR instruments: Bruker DPX400 (1H NMR: 400 MHz, 13C NMR: 100 MHz), Jeol ECX400 (1H NMR: 400 MHz, 13C NMR: 100 MHz), Jeol ECP 500 (1H NMR: 500 MHz, 13C NMR: 125 MHz), Bruker AVANCEIII500 (1H NMR: 500 MHz, 13C NMR: 125 MHz) or Bruker AVANCEIII700 (1H NMR: 700 MHz, 13C NMR: 175 MHz). All instruments were operated by the Core Facility BioSupraMol of the Freie Universität Berlin. Data processing was performed with MestReNova (v6.0.2-5475). Relative regioisomer proportions of [pG2]-based derivatives and [G2] OGD mixtures were analyzed by 13C NMR using sample concentrations of about 200 – 300 mg/mL.

3.2 HPLC

For analytical normal-phase HPLC analysis, a Nucleosil column from Macherey Nagel was used as stationary phase (pore size: 50 Å, particle size: 5 µm, length: 250 mm, diameter: 4 mm). Mixtures of *n*-hexane and isopropanol were used (*v*:*v*) as mobile phase. The NP HPLC system was equipped with a Smartline pump 1050, a Smartline UV detector 2550, and a Smartline RI detector 2300, which were purchased from Knauer. The system was operated with a flow rate of 1 mL/min. Data processing and analysis was performed with ChromeGate Client Viewer (*v*.3.3.2) from Knauer. For preparative normal-phase HPLC, a Nucleosil column from Macherey Nagel was used as stationary phase (pore size: 50 Å, particle size: 5 µm, length: 250 mm, diameter: 32 mm). The preparative HPLC system consisted of a Smartline pump 1800, UV variable wavelength monitor from Knauer, and a Smartline RI detector 2400. The system was operated with a flow rate of 64 mL/min. The normal-phase HPLC system was operated by Marleen Selent.

For analytical reversed-phase HPLC, a system from Knauer was used, which was equipped with two Smartline 1000 pumps, a variable wavelength UV detector 2500, RI detector, and an Autosampler 3950. As stationary phase, a pre-packed C18 column was used (RSC-Gel, C18ec, pore size: 100 Å, particle size: 5 µm, length: 250 mm, diameter: 4 mm). The flow rate was 1 mL/min. Data processing and analysis was performed with ChromeGate Client Viewer (*v*.3.3.2) from Knauer. For reversed-phase HPLC purification, a setup from Knauer was used, which consisted of a Smartline Manager 5000 (+ interface-module), two Smartline Pumps 1000, a 6-port-3-channel-injection valve, a sample loop (10 mL), UV Detektor 2500, RI detector, and a high pressure gradient mixer. Spectra were recorded with a x-y-plotter from Knauer. As stationary phase, a pre-packed column was used (RSC-Gel, C18ec, 5 µm). The setup was constructed by Dr. Carlo Fasting. The mobile phase was degassed prior usage. The flow rate was adjusted to 20 mL/min and the detection wavelength was 240 nm.

3.3 Synthesis

3.3.1 [pG1]-OH Regioisomer Mixture (a,b)

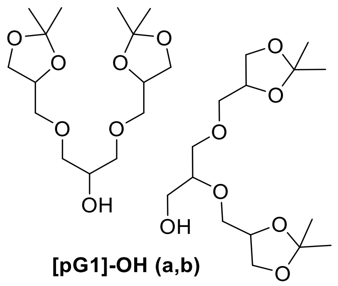

[pG1]-OH (a,b)

[pG1]-OH (a:b, 6:4).[1] Technical triglycerol (see Table S1) was dissolved in 2,2´-dimethoxypropane (3 eq.) and *p*-toluenesulfonic acid monohydrate (0.1 eq.) was added. The reaction mixture was stirred at 40 °C for 16 hours. Triethylamine (0.1 eq.) was added and the solvent was removed under reduced pressure. The raw material was purified by column chromatography (SiO_2, *n*-hexane/EtOAc, 2/1 → 1/6) and the **[pG1]-OH** regioisomer mixture was obtained as pale yellow oil (**a**:**b**, 6:4, ~ 60%). 1H NMR (400 MHz, MeOD-d_4) δ = 4.28 - 4.20 (m, 1.9 H), 4.06 - 3.99 (m, 1.9 H), 3.87 - 3.80 (m, 0.8 H), 3.75 - 3.44 (m, 11 H). 13C NMR (101 MHz, MeOD-d_4) δ = 110.4, 81.4, 76.3 - 76.1, 73.9, 73.4, 72.4 - 72.3, 70.6, 67.5, 62.6, 27.0, 25.6. MS (ESI): *m/z* = 343.1739 $C_{15}H_{28}O_7Na_1^+$ (calculated = 343.1727). Isomer ratios and product yields obtained from other oligoglycerol charges are summarized in Table 1 (see manuscript).

3.3.1 [pG1]-OH (a)

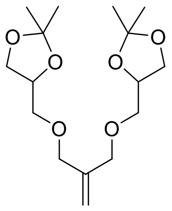

[pG1]-ene (a)

[pG1]-ene (a).[1a] DL-1,2-Isopropylidenglycerol (5.62 g, 42.5 mmol) was dissolved in THF (125 mL) and NaH (60w%, 5.10 g, 128 mmol) was added. Catalytic amounts of 15-crown-5 were added and the mixture was stirred at 50 °C for 1 h. Methallyl dichloride (2.65 g, 21.2 mmol), catalytic amounts of 18-crown-6, and potassium iodide were added and the mixture was stirred at 80 °C for 12 h. The reaction mixture was then allowed to cool down to room temperature and water (10 mL) was added drop wise. The solvent was removed under reduced pressure and the residue was suspended in water (200 mL) and Brine (100 mL). The aqueous layer was extracted with EtOAc (3 x 150 mL). The organic layer was dried over Na_2SO_4 and the solvent was removed under reduced pressure. Column chromatography (SiO_2, *n*-pentane/EtOAc, 8:1 → 2:1) led to the obtainment of **[pG1]-ene (a)** (3.75 g, 11.9 mmol, 56%). 1H NMR (400 MHz, MeOD-d_4) δ = 5.20 - 5.18 (m, 2H), 4.30 - 4.21 (m, 2H), 4.06 - 3.99 (m 6H), 3.75 - 3.69 (m, 2H), 3.52 - 3.43 (m, 4H), 1.42 - 1.31 (m, 12H). 13C NMR (101 MHz,

MeOD-d_4) δ = 144.1, 114.7, 110.5, 76.1, 72.9, 72.1, 67.6, 27.1, 25.7. MS (ESI): *m/z* = 339.1802 $C_{16}H_{28}O_6Na_1^+$ (calculated = 339.1778).

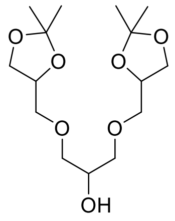

[pG1]-OH (a)

[pG1]-OH (a).[1a] The starting material **[pG1]-ene (a)** (2.75 g, 8.69 mmol) was dissolved in dry MeOH (20 mL) and dry DCM (20 mL). The reaction mixture was cooled down to -78 °C and ozone was guided through the mixture until its colour changed to deep blue. Oxygen was then guided through the mixture for another 30 minutes and sodium borohydride was added (3.28 g, 86.9 mmol). The mixture was allowed to warm up to room temperature overnight before a saturated aqueous solution of NH_4Cl (35 mL) was added. The aqueous layer was extracted with DCM (6 x 50 mL). The organic layer was dried over Na_2SO_4 and the solvent was removed under reduced pressure. Column chromatography (SiO_2, DCM/EtOAc, 97/3 + 3% MeOH) gave the desired product **[pG1]-OH (a)** (2.20 g, 6.86 g, 79%). 1H NMR (400 MHz, MeOD-d_4) δ = 4.30 - 4.22 (m, 2H), 4.09 - 4.02 (m, 2H), 3.90 - 3.82 (m, 1H), 3.76 - 3.70 (m, 2H), 3.59 - 3.46 (m, 8H), 1.42 - 1.30 (m, 12H). 13C NMR (101 MHz, MeOD-d_4) δ = 110.4, 76.1, 73.9, 73.4, 70.5, 67.5, 27.0, 25.6. MS (ESI): *m/z* = 343.1726 $C_{15}H_{28}O_7Na_1^+$ (calculated = 343.1727).

3.3.2 [pG1]-OH (b)

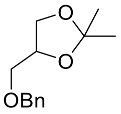

[pG0]-OBn

[pG0]-OBn. D,L-Isopropylidenglycerol (2.00 g, 15.1 mmol) was dissolved in DMF (80 mL) and the flask was cooled in an mixture of water and ice (50:50). NaH (60w%, 2.42 g, 60.5 mmol) was added in small portions, while the mixture was stirred and allowed to warm up to room temperature. Benzyl bromide (3.10 g, 18.2 mmol) was added and the mixture was stirred for 12 hours at room temperature. Subsequently, water (10 mL) was added drop wise and the solvent was removed under reduced pressure. The remaining material was suspended with Brine (200 mL) and water (100 mL) and the aqueous layer was extracted with EtOAc (3 x 150 mL). The combined organic layers were dried over Na_2SO_4 and the solvent was removed under reduced pressure. Column chromatography (SiO_2, *n*-pentane/EtOAc, 10/1 → 8/1) gave the desired product **[pG0]-OBn** (2.10 g, 9.45 mmol, 63%). 1H NMR (400 MHz, MeOD-d_4) δ = 7.36 - 7.25 (m, 5H), 4.56 - 4.55 (s, 2H), 4.30 - 4.22 (m, 1H), 4.07 - 4.00 (m, 1H), 3.75 - 3.68 (m, 1H), 3.55 - 3.46 (m, 2H), 1.40 - 1.29 (m, 6H). 13C NMR (101 MHz,

MeOD-d$_4$) δ = 139.5, 129.4, 128.8, 128.7, 110.5, 76.1, 74.4, 72.1, 67.6, 27.0, 25.7. MS (ESI): *m/z* = 245.1139 $C_{13}H_{18}O_3Na_1^+$ (calculated = 245.1148).

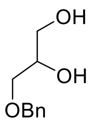

[G0]-OBn

[G0]-OBn (b). The starting material **[pG0]-OBn (b)** (2.10 g, 9.45 mmol) was dissolved in MeOH (300 mL) and HCl (37%, 100 µL) was added. The mixture was stirred for two hours at room temperature and the solvent was removed under reduced pressure. The procedure was repeated and the solvent was removed under reduced pressure to obtain the desired product **[G0]-OBn (b)** (1.72 g, 9.43 mmol, 99%). 1H NMR (400 MHz, MeOD-d$_4$) δ = 7.36 - 7.22 (m, 5H), 4.53 (s, 2H), 3.84 - 3.77 (m, 1H), 3.64 - 3.45 (m, 4H). 13C NMR (101 MHz, MeOD-d$_4$) δ = 139.6, 129.3, 128.8, 128.6, 74.3, 72.7, 72.2, 64.5. MS (ESI): *m/z* = 205.0832 $C_{10}H_{14}O_3Na_1^+$ (calculated = 205.0835).

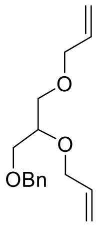

[G0-(ene)$_2$]-OBn

[G0-(ene)$_2$]-OBn (b). The starting material **[G0]-OBn (b)** (0.50 g, 2.74 mmol) was dissolved in THF (50 mL). NaH (60w%, 0.55 g, 13.7 mmol) and catalytic amounts of 15-crown-5 were added. The mixture was stirred at 50 °C for 1 hour. Allyl bromide (712 µL, 8.32 mmol), catalytic amounts of 18-crown-6, and potassium iodide were added. The mixture was stirred at 80 °C for 12 hours and water (10 mL) was added drop wise. The solvent was removed under reduced pressure and the residue was suspended in water (50 mL) and Brine (50 mL). The aqueous layer was extracted with DCM (5 x 50 mL), the organic layer was dried over Na_2SO_4, and the solvent was removed under reduced pressure. Column chromatography (SiO_2, pentane/DCM, 1/1 → 0/1) gave **[G0-(ene)$_2$]-OBn (b)** (0.56 g, 2.13 mmol, 78%). 1H NMR (400 MHz, MeOD-d$_4$) δ = 7.36 - 7.23 (m, 5H), 5.98 - 5.80 (m, 2H), 5.33 - 5.21 (m, 2H), 5.19 - 5.09 (m, 2H), 4.52 (s, 2H), 4.16 - 4.08 (m, 2H), 4.02 - 3.92 (m, 2H), 3.73 - 3.66 (m, 1H), 3.61 - 3.48 (m, 4H). 13C NMR (101 MHz, MeOD-d$_4$) δ = 139.6, 136.4, 136.0, 129.4, 128.8, 128.7, 117.1, 78.5, 74.3, 73.2, 72.2, 71.1. MS (ESI): *m/z* = 285.1473 $C_{16}H_{22}O_3Na_1^+$ (calculated = 285.1461).

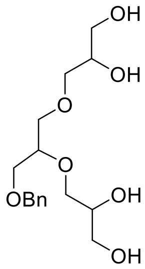

[G0]-OBn (b)

[G1]-OBn (b). The starting material **[G0-(ene)$_2$]-OBn (b)** (7.29 g, 29.4 mmol) was dissolved in a mixture of degassed water (400 mL) and ^{t}BuOH (400 mL). TMANO (9.80 g, 130 mmol), citric acid (28.2 g, 146 mmol), and $K_2OsO_4*2H_2O$ (1.35 g, 3.67 mmol) were added. The mixture was stirred for 12 hours at room temperature, the ion exchange resin Lewatit K 6267 (92.0 g) was added, and the mixture was stirred again for one hour. The resin was filtered off, washed with water (400 mL) and the solvent was removed under reduced pressure. Column chromatography (SiO_2, DCM/MeOH, 9/1 → 2/1) led to the desired product **[G1]-OBn (b)** (3.37 g, 10.2 mmol, 35%). 1H NMR (400 MHz, MeOD-d_4) δ = 7.42 - 7.23 (m, 5H), 5.20 (s, 4H), 4.53 (s, 2H), 3.84 - 3.66 (m, 4H), 3.64 - 3.41 (m, 11H). 13C NMR (101 MHz, MeOD-d_4) δ = 139.5, 129.2, 128.8, 128.5, 79.5, 74.2, 73.9, 73.6, 72.6, 72.2 - 72.1, 71.9, 70.9, 64.2. MS (ESI): *m/z* = 353.1573 $C_{16}H_{26}O_7Na_1^+$ (calculated = 353.1571).

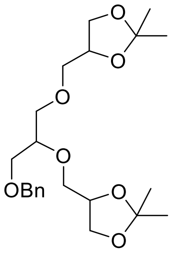

[pG0]-OBn (b)

[pG1]-OBn (b). The starting material **[G1]-OBn (b)** (2.82 g, 8.54 mmol) was dissolved in 2,2´-dimethoxypropane (200 mL) and *p*-toluenesulfonic acid monohydrate (0.30 g, 1.55 mmol) was added. The mixture was stirred for one hour at room temperature and triethylamine (110 µL, 1.55 mmol) was added. Column chromatography (SiO_2, DCM/EtOAc, 1/0 → 4/1) gave the product **[pG1]-OBn (b)** (3.00 g, 7.31 mmol, 86%). 1H NMR (400 MHz, MeOD-d_4) δ = 7.36 - 7.24 (m, 5H), 4.53 (s, 2H), 4.26 - 4.18 (m, 2H), 4.05 - 3.99 (m, 2H), 3.77 - 3.47 (m, 11H), 1.41 - 1.28 (m, 12H). 13C NMR (101 MHz, MeOD-d_4) δ = 139.6, 129.3, 128.8, 128.6, 110.4, 79.8, 76.2 - 76.1, 74.3, 73.3, 72.5 - 72.3, 71.0, 67.7, 67.5, 27.1, 25.6. MS (ESI): *m/z* = 433.2210 $C_{22}H_{34}O_7Na_1$+ (calculated = 433.2197).

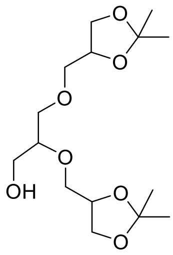

[pG0]-OH (b)

[pG1]-OH (b). The starting material **[pG1]-OBn (b)** (0.98 g, 2.39 mmol) was dissolved in a mixture of cyclohexane (5 mL) and THF (5 mL). Pd/C (10w%, 50 mg) was added and the mixture was stirred under hydrogen atmosphere (5 bar) for 12 hours. The mixture was passed through a syringe filter (0.20 µm, RC) and the solvent was removed under reduced pressure. Column chromatography (SiO_2, *n*-pentane/EtOAc, 1/0 → 1/4) gave the desired product **[pG1]-OH (b)** (0.66 g, 2.03 mmol, 85%). 1H NMR (400 MHz, MeOD-d_4) δ = 4.29 - 4.21 (m, 2H), 4.08 - 4.02 (m, 2H), 3.79 - 3.48 (m, 11H), 1.42 - 1.28 (m, 12H). 13C NMR (101 MHz, MeOD-d_4) δ = 110.2, 81.3, 76.1, 75.9, 73.2, 72.3 - 72.1, 67.6, 67.4, 62.5, 27.1, 25.7. MS (ESI): *m/z* = 343.1739 $C_{15}H_{28}O_7Na_1^+$ (calculated = 343.1727).

3.3.3 [pG2]-OH Regioisomer Mixture (aa,ab,bb)

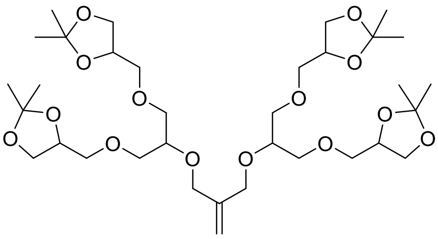

[pG2]-ene (aa)

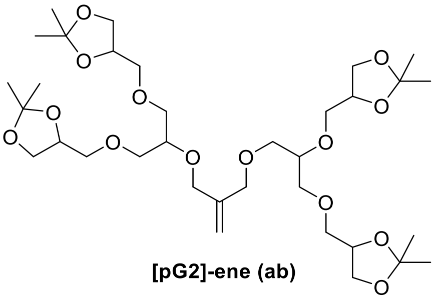

[pG2]-ene (ab)

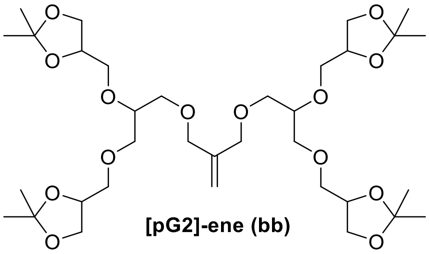

[pG2]-ene (bb)

[pG2]-ene (aa,ab,bb).[1] The starting material **[pG1]-OH** (13.6 g, 42.5 mmol, **a**:**b**, 6:4) was dried under reduced pressure (~ 10^{-2} mbar), dissolved in dry THF (125 mL), and NaH (60w%, 5.10 g, 128 mmol) was added in small portions. 15-Crown-5 (catalytic amounts) was added and the mixture was stirred for 45 minutes at 50 °C. Methallyl dichloride (2.46 mL, 21.2 mmol), potassium iodide (catalytic amounts), and 18-crown-6 (catalytic amounts) were added. The reaction was stirred at 70 °C for 24 hours. The flask was then cooled with an ice bath before water (80 mL) was added slowly. The solvent was removed under reduced pressure and the crude product was suspended with Brine (200 mL) and DCM (150 mL). The aqueous layer was extracted with DCM (3 x 150 mL). The organic

layer was dried over Na_2SO_4 and the solvent was removed under reduced pressure. Column chromatography (SiO_2, *n*-pentane/EtOAc, 1/2 → 0/1) gave the desired product **[pG2]-ene** (12.2 g, 17.6 mmol, **aa**:**ab**:**bb**, 4:4:1, 83%).1H NMR (400 MHz, MeOD-d_4): δ = 5.22 - 2.21 (m, 2H), 4.29 - 4.22 (m, 4H), 4.19 - 4.13 (m, 2H), 4.08 - 4.01 (m, 4H), 3.79 - 3.46 (m, 24 H), 1.40 - 1.30 (m, 24H). 13C NMR (101 MHz, MeOD-d_4): δ = 145.2, 144.8, 114.5, 110.4, 79.9, 78.4, 76.1, 73.4, 72.9, 72.4 - 72.3, 71.6, 71.1, 67.7 - 67.6, 27.1, 25.7. MS (ESI): *m/z* = 715.3982 $C_{34}H_{60}O_{14}Na_1^+$ (calculated = 715.3875).

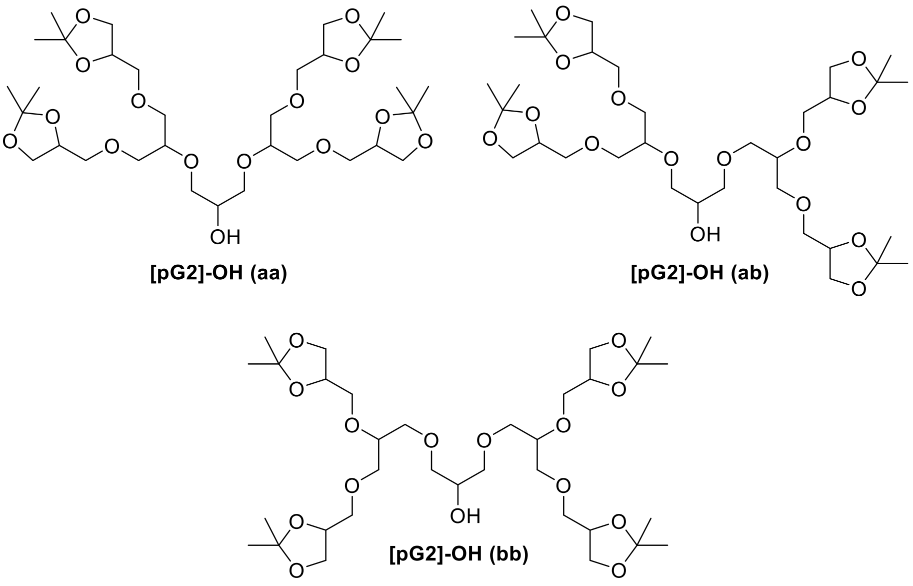

[pG2]-OH (aa) **[pG2]-OH (ab)**

[pG2]-OH (bb)

[pG2]-OH (aa,ab,bb).[1] The starting material **[pG2]-ene** (8.00 g, 11.5 mmol, **aa**:**ab**:**bb**, 4:4:1) was dried was dried under reduced pressure (~ 10^{-2} mbar) and dissolved in a mixture of dry DCM (35 mL) and dry MeOH (35 mL). The mixture was cooled down to -78 °C and ozone was passed through the reaction mixture until its colour changed to deep blue. Oxygen was then passed through the solution until it became colourless. Sodium borohydride (4.36 g, 115 mmol) was added slowly and the mixture was allowed to heat up to RT overnight. A saturated aqueous solution of NH_4Cl (100 mL) was added and the mixture was extracted with DCM (5 x 50 mL). The organic layer was dried over Na_2SO_4 and the solvent was removed under reduced pressure. Column chromatography (SiO_2, DCM/EtOAc, 97/3 + 3% MeOH) gave the desired product (6.40 g, 9.18 mmol, **aa**:**ab**:**bb**, 4:4:1, 80%). 1H NMR (400 MHz, MeOD-d_4): δ = 4.29 - 4.21 (m, 4H), 4.08 - 4.02 (m, 4H), 3.80 - 3.47 (m, 27H), 1.42 - 1.31 (m, 24H). 13C NMR (101 MHz, MeOD-d_4): δ = 109.1, 78.5, 74.8, 72.5 - 72.1, 71.4 - 71.1, 69.8 - 69.5, 66.2, 25.8, 24.3. MS (ESI): *m/z* = 719.3920 $C_{33}H_{60}O_{15}Na_1^+$ (calculated = 719.3824).

3.3.3 [pG2]-OH (aa)

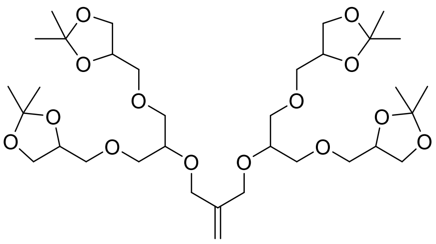

[pG2]-ene (aa)

[pG2]-ene (aa).[1a] The starting material **[pG1]-OH (a)** (1.00 g, 3.12 mmol) was dried under reduced pressure (~ 10^{-2} mbar), dissolved in dry THF (60 mL), and NaH (60w%, 0.18 g, 4.68 mmol) was added. 15-Crown-5 (catalytic amounts) was added and the mixture was stirred for one hour at 50 °C. Methallyl dichloride (0.17 mL, 1.48 mmol), potassium iodide (catalytic amounts), and 18-crown-6 (catalytic amounts) were added. The reaction was stirred at 80 °C for 12 hours. The reaction mixture was allowed to cool down to room temperature and water (6 mL) was added drop wise. The solvent was removed under reduced pressure and the crude product was mixed with water (60 mL), Brine (60 mL), and DCM (60 mL). The aqueous layer was extracted with EtOAc (6 x 50 mL). The organic layer was dried over Na_2SO_4 and the solvent was removed under reduced pressure. Column chromatography (SiO_2, *n*-pentane/EtOAc, 8/1 → 4/1 + 3% MeOH) gave the desired product **[pG2]-ene (aa)** (0.81 g, 1.17 mmol, 37%). 1H NMR (400 MHz, MeOD-d_4): δ = 5.20 (s, 2H), 4.27 - 4.21 (m, 4H), 4.17 (s, 4H), 4.07 - 4.02 (m, 4H) 3.76 - 3.71 (m, 4H), 3.67 - 3.47 (m, 18H), 1.58 - 1.16 (m, 24H). 13C NMR (101 MHz, MeOD-d_4): δ = 144.9, 114.2, 110.0, 98.9, 78.2, 75.7, 73.2, 72.2, 71.4, 67.4, 63.1, 27.2, 25.9. MS (ESI): *m/z* = 715.3889 $C_{34}H_{60}O_{14}Na_1^+$ (calculated = 715.3875).

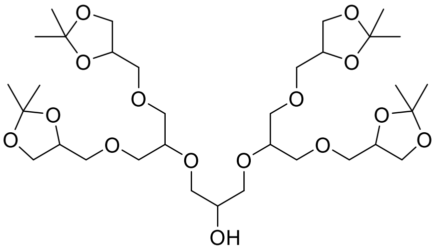

[pG2]-OH (aa)

[pG2]-OH (aa).[1a] The starting material **[pG2]-ene (aa)** (0.81 g, 1.22 mmol) was dried was dried under reduced pressure (~ 10^{-2} mbar) and dissolved in a mixture of dry DCM (40 mL) and dry MeOH (20 mL). The mixture was cooled down to -78 °C and ozone was passed through it until its colour changed to deep blue. Ozone was then passed through it for another 30 minutes. Sodium borohydride (0.46 g, 12.2 mmol) was added slowly and the mixture was allowed to warm up to room temperature overnight. A saturated aqueous solution of NH_4Cl (30 mL) was added and the mixture was extracted with EtOAc (6 x 30 mL). The organic layer was dried over Na_2SO_4 and the solvent was removed under reduced pressure. Column chromatography (SiO_2, *n*-pentane/EtOAc, 1/1 + 3% MeOH) gave the desired product (140 mg, 190 µmol, 15%). 1H NMR (400 MHz, MeOD-d_4): δ = 4.28 - 4.21 (m, 4H),

4.08 - 4.03 (m, 4H), 3.88 - 3.79 (m, 1H), 3.75 - 3.51 (m, 26H), 1.49 - 1.22 (m, 24H). 13C NMR (101 MHz, MeOD-d$_4$): δ = 110.3, 79.6, 75.9, 73.3, 72.6, 72.3 - 72.2, 71.0, 67.5, 27.1, 25.7. MS (ESI): *m/z* = 719.3813 $C_{33}H_{60}O_{15}Na_1^+$ (calculated = 719.3824).

3.3.4 [pG2]-OH (bb)

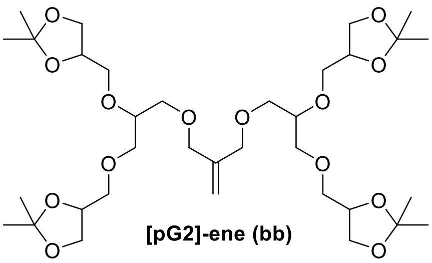

[pG2]-ene (bb)

[pG2]-ene (bb). The starting material **[pG1]-OH (b)** (0.65 g, 2.14 mmol) was dried under reduced pressure (~ 10^{-2} mbar), dissolved in dry THF (60 mL), and NaH (60w%, 0.13 g, 3.21 mmol) was added. 15-Crown-5 (catalytic amounts) were added and the mixture was stirred for one hour at 50 °C. Methallyl dichloride (0.11 mL, 1.02 mmol), potassium iodide (catalytic amounts), and 18-crown-6 (catalytic amounts) were added. The reaction was stirred at 80 °C for 12 hours. The reaction mixture was allowed to cool down to room temperature and water (10 mL) was added drop wise. The solvent was removed under reduced pressure and the crude product was mixed with water (60 mL), Brine (60 mL), and DCM (60 mL). The aqueous layer was extracted with EtOAc (6 x 50 mL). The organic layer was dried over Na_2SO_4 and the solvent was removed under reduced pressure. Column chromatography (SiO_2, *n*-pentane/EtOAc, 8/1 → 4/1 + 3% MeOH) gave the product **[pG2]-ene (bb)** (0.20 g, 0.30 mmol, 14%). 1H NMR (400 MHz, MeOD-d$_4$): δ = 5.15 (s, 2H), 4.24 - 4.16 (m, 4H), 4.04 - 3.95 (m, 8H), 3.75 - 3.41 (m, 22H), 1.38 - 1.16 (m, 24H). 13C NMR (101 MHz, MeOD-d$_4$): δ = 144.3, 114.6, 110.4, 79.9, 76.1, 73.4, 72.9, 72.4, 71.2, 67.8, 67.6, 49.0, 27.2, 25.7. MS (ESI): *m/z* = 715.3857 $C_{34}H_{60}O_{14}Na_1^+$ (calculated = 715.3875).

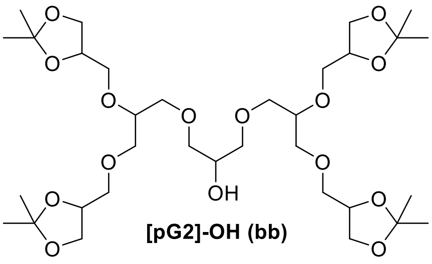

[pG2]-OH (bb)

[pG2]-OH (bb). The starting material **[pG2]-ene (bb)** (220 mg, 0.32 mmol) was dried was dried under reduced pressure (~ 10^{-2} mbar) and dissolved in a mixture of dry DCM (35 mL) and dry MeOH (20 mL). The mixture was cooled down to -78 °C and ozone was passed through it until its colour changed to deep blue. Oxygen was then passed through it until the mixture became colourless. Sodium borohydride (0.37 g, 9.78 mmol) was added slowly and the mixture was allowed to warm up to room temperature overnight. A saturated aqueous solution of NH_4Cl (17 mL) was added and the

mixture was extracted with EtOAc (5 x 30 mL). The solvent was removed under reduced pressure and column chromatography (SiO_2, *n*-pentane/EtOAc, 1/1 → 1/1 + 3% MeOH) gave the desired product (150 mg, 0.22 mmol, 67%). 1H NMR (400 MHz, MeOD-d_4): δ = 4.28 - 4.21 (m, 4H), 4.07 - 4.02 (m, 4H), 3.88 - 3.83 (m, 1H), 3.78 - 3.45 (m, 27H), 1.62 - 1.11 (m, 24 H). 13C NMR (101 MHz, MeOD-d_4): δ = 110.4, 79.8, 76.1, 73.9, 73.4, 72.4, 70.6, 67.7, 67.6, 27.2, 25.7. MS (ESI): *m/z* = 719.3830 $C_{33}H_{60}O_{15}Na_1^+$ (calculated = 719.3824).

3.3.5 [G1] OGD Regioisomer Mixture 2

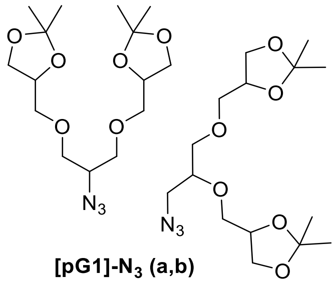

[pG1]-N$_3$ (a,b)

[pG1]-N$_3$ (a:b, 6:4). The starting material **[pG1]-OH** (5.50 g, 17.1 mmol, **a**:**b**, 6:4) was dissolved in toluene (150 mL). Triethylamine (2.66 mL, 20.6 mmol) and methanesulfonyl chloride (1.59 mL, 20.6 mmol) were added. The mixture was stirred for 16 hours at room temperature, the precipitate was filtered off, and the solvent was removed under reduced pressure. The obtained raw material (7.23 g) was dissolved in DMF (100 mL), NaN_3 (5.89 g, 90.7 mmol) was added, and the mixture was stirred for 16 hours at 80 °C. The excess of NaN_3 was filtered off using a filter paper and the solvent was removed under reduced pressure. Column purification (SiO_2, *n*-hexane/isopropanol, 15:1) led to the obtainment of **[pG1]-N$_3$** (4.76 g, 13.8 mmol, **a**:**b**, 6:4, 81%). 1H NMR (700 MHz, MeOD-d_4): δ = 4.27 - 4.22 (m, 2H), 4.08 - 4.02 (m, 2H), 3.80 - 3.49 (m, 10.3H), 3.43 - 3.31 (m, 0.7H), 1.44 - 1.27 (m, 12H). 13C NMR (175 MHz, MeOD-d_4): δ = 110.4, 79.9, 76.0, 73.4 - 73.1, 72.3 - 72.0, 67.0 - 67.4, 61.9, 52.8, 27.1, 25.6.

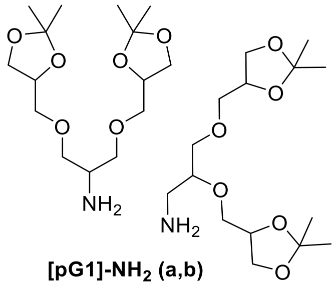

[pG1]-NH$_2$ (a,b)

[pG1]-NH$_2$ (a:b, 6:4). The starting material **[pG1]-N$_3$** (2.06 g, 5.96 mmol, **a**:**b**, 6:4) was dissolved in MeOH (20 mL), Pd/C (200 mg) was added, and the mixture was stirred under hydrogen atmosphere (5 bar) for 12 hours at room temperature. The mixture was passed through a syringe filter (0.2 µm, RC). The solvent was removed under reduced pressure to obtain **[pG1]-NH$_2$** (1.56 g, 4.88 mmol, **a**:**b**, 6:4, 82%). 1H NMR (700 MHz, MeOD-d_4): δ = 4.97 (s, 2H), 4.52 - 4.47 (m, 2H), 4.33 - 4.25 (m, 2H),

4.02 - 3.64 (m, 10H), 3.02 - 2.88 (m, 0.8H), 1.69 - 1.50 (m, 12H). 13C NMR (175 MHz, MeOD-d4): δ = 110.4, 81.5 - 81.4, 76.4 - 76.1, 74.0, 73.3 - 73.0, 72.6, 72.0, 67.5, 51.7, 43.6, 27.0, 25.6. MS (ESI): *m/z* = 432.1881 $C_{15}H_{29}N_{1}O_{6}Na_{1}^{+}$ (calc. = 342.1887).

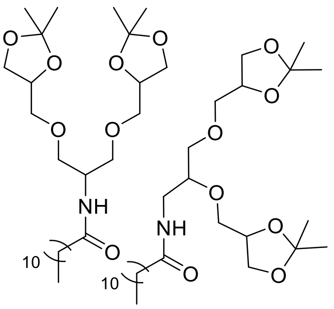

[pG1]-amide-C12 (a,b)

[pG1]-amide-C12 (a:b, 6:4). *N*-Hydroxysuccinimide (0.86 g, 7.52 mmol) and dodecanoic acid (1.25 g, 6.26 mmol) were dissolved in dry DCM (30 mL). The mixture was cooled with an ice bath, *N,N'*-dicyclohexylcarbodiimide was added (1.55 g, 7.52 mmol). The mixture was stirred for one hour and allowed to warm up to room temperature. **[pG1]-NH2** (2.00 g, 6.26 mmol, **a**:**b**, 6:4) was added and the mixture was stirred for another 16 hours at room temperature. The precipitate was filtered off using a filter paper and the solvent was removed under reduced pressure. Column chromatography (SiO_2, DCM/EtOAc, 4/1 → 4/1 + 3% MeOH) gave the product **[pG1]-amide-C12** (1.52 g, 3.00 mmol, **a**:**b**, 6:4, 48%). 1H NMR (400 MHz, MeOD-d4): δ = 4.86 (s, 1H), 4.30 - 4.21 (m, 2.3H), 4.20 - 4.13 (0.7H), 4.08 - 4.01 (m, 2H), 3.77 - 3.46 (m, 10H), 2.24 - 2.16 (m, 2H), 1.67 - 1.54 (m, 2H), 1.45 - 1.22 (m, 28H), 0.95 - 0.87 (m, 3H). 13C NMR (101 MHz, MeOD-d4) δ = 176.0, 174.7, 110.9 - 110.4, 79.2 - 79.0, 76.3 - 76.0, 73.4 - 72.9, 72.4, 72.1 - 72.0, 71.2 - 71.4, 67.5 - 67.4, 50.0, 41.3, 37.0, 33.0, 30.7 - 30.2, 27.1 - 27.0, 26.2, 25.6, 23.7, 14.4. MS (ESI): *m/z* = 524.3561 $C_{27}H_{51}N_{1}O_{7}Na_{1}^{+}$ (calculated = 524.3558).

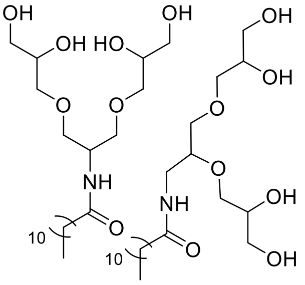

[G1]-amide-C12 (a,b)

[G1]-amide-C12 (a:b, 6:4). The starting material **[pG1]-amide-C12** (1.52 g, 3.00 mmol, **a**:**b**, 6:4) was dissolved in MeOH and HCl (37%, 100 µL) was added. The mixture was stirred for 12 hours at room temperature and the solvent was removed under reduced pressure. The raw product was dissolved in in a mixture of water and MeOH (1/1, *v/v*, 20 mL) and passed through a syringe filter (RC, 0.2 µm). Subsequent purification by reversed-phase HPLC (water/MeOH, 3/7) led to the obtainment of **[G1]-amide-C12** (758 mg, 1.80 mmol, **a**:**b**, 6:4, 60%). 1H NMR (400 MHz, MeOD-d4): δ = 4.21 - 4.12

(m, 0.7H), 3.80 - 3.70 (m, 2.3H), 3.66 - 3.40 (m, 12H), 2.24 - 2.17 (m, 2H), 1.66 - 1.55 (m, 2H), 1.40 - 1.20 (m, 16H), 0.95 - 0.85 (m, 3H). 13C NMR (101 MHz, MeOD-d4) δ = 176.3 - 176.0, 79.0, 73.7 - 73.3, 72.5 - 71.9, 71.0 - 70.9, 64.1, 50.0, 41.1 - 41.0, 36.9, 32.9, 30.6 - 30.2, 26.9, 23.6, 14.5. MS (ESI): *m/z* = 444.2944 $C_{21}H_{43}N_1O_7Na_1^+$ (calculated = 444.2932).

3.3.6 [G1] OGD Regioisomer Mixture 4

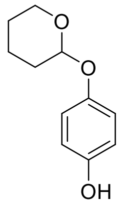

THP-O-hyd-OH

THP-O-hyd-OH. Hydroquinone (5.00 g, 45.4 mmol) was dissolved in a mixture of DCM (150 mL) and THF (50 mL). 3,4-Dihydro-2*H*-pyran (4.97 mL, 54.5 mmol) and *p*-toluenesulfonic acid monohydrate (0.86 g, 4.54 mmol) were added. The mixture was stirred at room temperature and the course of the reaction was followed by TLC (SiO_2, DCM). After 16 hours, triethylamine (1.26 mL, 9.08 mmol) and DCM (150 mL) were added. The organic layer was washed with water and dried over Na_2SO_4. The solvent was removed under reduced pressure. Column chromatography (SiO_2, DCM/EtOAc, 1/0 → 9/1) led to the obtainment of **THP-O-hyd-OH** (3.35 g, 17.2 mmol, 38%). 1H NMR (MeOD-d4, 400 MHz, ppm): δ = 6.86 - 6.84 (m, 2H), 6.67 - 6.65 (m, 2H), 5.21 - 5.19 (t, 2H), 3.94 - 3.88 (m, 1H), 3.57 - 3.52 (m, 1H); 2.00 - 1.54 (m, 6H). 13C NMR ($CDCl_3$, 100 MHz, ppm): δ = 151.9, 150.3, 117.7, 115.3, 97.7, 61.9, 48.1, 47.9, 47.7, 47.5, 47.2, 47.0, 30.4, 25.1, 18.9. (ESI): *m/z* = 217.0859 $C_{11}H_{14}O_3Na^+$ (calculated = 217.0835).

THP-O-hydC4

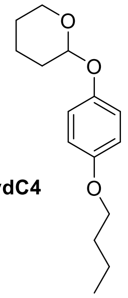

THP-O-hydC4. The starting material **THP-O-hyd-OH** (0.60 g, 3.09 mmol) and potassium carbonate (0.51 g, 3.71 mmol) were dissolved in dry DMF (40 mL). 1-Bromobutane (0.40 mL, 3.71 mmol) was added and the mixture was stirred at 85 °C for 16 hours. The mixture was allowed to cool down to room temperature before water (25 mL) and a saturated aqueous solution of NH_4Cl (25 mL) were added. The aqueous layer was extracted with EtOAc (5 x 50 mL). The organic layers were washed with water and dried over Na_2SO_4. The solvent was removed under reduced pressure. Column chromatography (SiO_2, DCM) led to the obtainment of **THP-O-hydC4** (0.55 g, 2.19 mmol, 56%). 1H NMR (acetone-d6, 400 MHz, ppm): δ = 6.99 - 6.93 (m, 2H), 6.86 - 6.80 (m, 2H), 5.31 - 5.26 (m,

1H), 3.95 - 3.83 (m, 3H), 3.57 - 3.50 (m, 1H), 2.10 - 1.43 (m, 10H), 1.00 - 0.93 (m, 3H). 13C NMR (acetone-d_6, 100 MHz, ppm): δ = 155.0, 152.0, 118.5, 115.8, 97.9, 68.5, 62.3, 32.2, 31.2, 26.0, 19.9, 19.6, 14.1. (ESI): m/z = 273.1484 $C_{15}H_{22}O_3Na^+$ (calculated = 273.1461).

HO-hydC4

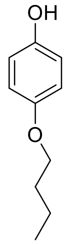

HO-hydC4. The starting material **THP-O-hydC4** (550 mg, 2.19 mmol) was dissolved in degassed MeOH (50 mL) and Dowex® 50WX8 (5 g) was added. The mixture was stirred at room temperature for two hours and the reaction was monitored by TLC (SiO_2, DCM). The solvent was removed under reduced pressure to obtain the desired product **HO-hydC4** (350 mg, 2.10 mmol, 96%). 1H NMR (MeOD-d_4, 400 MHz, ppm): δ = 6.76 – 6.66 (m, 4H), 3.87 (t, 2H), 1.69 (p, 2H), 1.48 (sext., 2H), 0.97 (t, 3H). 13C NMR ($CDCl_3$, 100 MHz, ppm): δ = 153.4, 149.4, 116.2, 115.7, 68.5, 31.5, 19.3, 14.0.

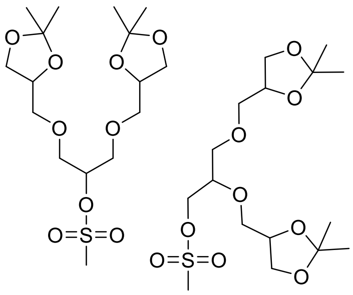

[pG1]-OMes (a,b)

[pG1]-OMes. The starting material **[pG1]-OH** (1.18 g, 3.71 mmol, **a**:**b**, 6:4) was dissolved in toluene (65 mL) and triethylamine (0.77 mL, 5.56 mmol) was added. Methanesulfonic acid (0.35 mmol, 4.63 mmol) was added and the mixture was stirred at room temperature for 16 hours. The precipitate was filtered off using a filter paper. The solvent of the filtrate was removed under reduced pressure to obtain **[pG1]-OMes** (1.47 g, 3.71 mmol, quant., **a**:**b**). The material was used without further purification.

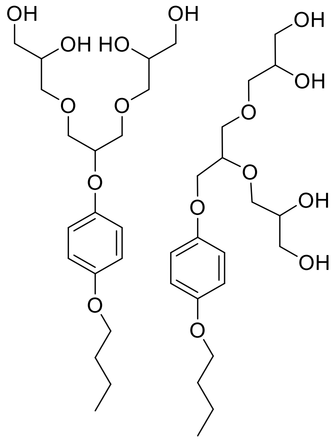

[G1]-ether-hydC4 (a,b)

[G1]-ether-hydC4 (a:b). The starting material **[pG1]-OMes** (839 mg, 2.10 mmol, **a**:**b**) was mixed with potassium carbonate (349 mg, 2.52 mmol) and **HO-hydC4** (350 mg, 2.10 mmol). Dry DMF (30 mL) was added and the mixture was stirred at 120 °C for 16 hours. The mixture was allowed to cool down to room temperature. Water (20 mL) and a saturated aqueous solution of NH_4Cl (20 mL) were added. The mixture was extracted with EtOAc (6 x 50 mL). The organic phases were washed with water and dried over Na_2SO_4. The solvent was removed under reduced pressure. Column chromatography (SiO_2, DCM/EtOAc, 1/0 → 4/1) gave the acetal-protected product (410 mg, 0.87 mmol, **a**:**b**). The material was dissolved in MeOH (50 mL) and Dowex® 50WX8 (5 g) was added. The mixture was stirred for 16 hours at room temperature. The mixture was filtered through filter paper. The solvent of the filtrate was removed under reduced pressure and the procedure was repeated to obtain **[G1]-ether-hydrC4** (260 mg, 0.66 mmol, **a**:**b**, 6:4, 31%). Purification by reversed-phase HPLC (ACN/water, 3/7) led to the obtainment of the individual regioisomers **a** and **b**. Characterization of regioisomer **a**: 1H NMR (MeOD-d_4, 400 MHz, ppm): δ = 6.97 – 6.91 (m, 2H), 6.86 – 6.79 (m, 2H), 4.47 – 4.40 (m, 1H), 3.95 – 3.88 (m, 2H), 3.79 – 3.65 (m, 6H), 3.61 – 3.45 (m, 8H), 1.76 – 1.66 (m, 2H), 1.55 – 1.43 (m, 2H), 1.01 – 0.93 (m, 3H). 13C NMR (MeOD-d_4, 100 MHz, ppm): δ = 155.4, 153.3, 119.0, 116.3, 79.1, 74.0, 72.1, 71.6, 69.2, 64.4. (ESI): m/z = 411.2036 $C_{19}H_{32}O_8Na^+$ (calculated = 411.1989). Characterization of regioisomer **b**: 1H NMR (MeOD-d_4, 400 MHz, ppm): δ = 6.89 – 6.80 (m, 4H), 4.09 – 3.96 (m, 2H), 3.93 – 3.83 (m, 3H), 3.81 – 3.47 (m, 12 H), 1.76 – 1.67 (m, 2H), 1.54 – 1.43 (m, 2H), 1.00 – 0.94 (m, 3H). 13C NMR (MeOD-d_4, 100 MHz, ppm): δ = 154.9, 154.2, 116.6, 116.4, 79.4, 74.0, 73.1, 72.9, 72.5, 72.3, 72.1, 69.7, 69.2, 64.3, 32.6, 20.3, 14.2. (ESI): m/z = 411.2031 $C_{19}H_{32}O_8Na^+$ (calculated = 411.1989).

3.3.7 [G2] OGD Regioisomer Mixture 6

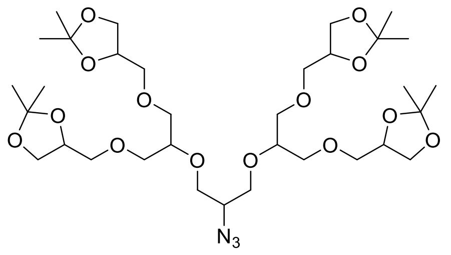

[pG2]-N$_3$ (aa)

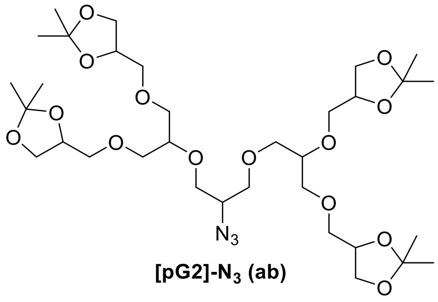

[pG2]-N$_3$ (ab)

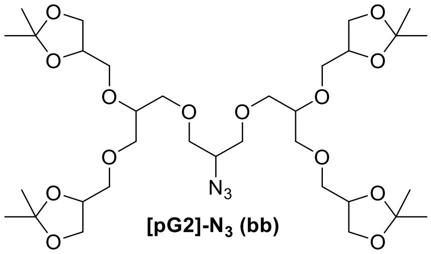

[pG2]-N$_3$ (bb)

[pG2]-N$_3$ (aa:ab:bb, 3:4:1).[1] The starting material **[pG2]-OH** (1.50 g, 2.15 mmol, **aa**:**ab**:**bb**, 4:4:1) was dissolved in toluene (100 mL). Triethylamine (0.41 mL, 3.22 mmol) was added, and methanesulfonyl chloride (0.22 mL, 2.78 mmol) was added. The mixture was stirred for 16 hours at room temperature before the precipitate was filtered off using a filter paper. The solvent was removed under reduced pressure. The so-obtained raw material was dissolved in DMF (100 mL), NaN3 (0.70 g, 10.7 mmol) was added, and the mixture was stirred for two hours at 120 °C. The excess of NaN3 was filtered off using a filter paper. The solvent was removed under reduced pressure. Column purification (SiO_2, DCM/EtOAc, 4:1 → 3:1) led to the obtainment of **[pG2]-N$_3$** (0.98 g, 1.35 mmol, **aa**:**ab**:**bb**, 3:4:1, 63%). 1H NMR (700 MHz, MeOD-d$_4$): δ = 4.27 - 4.22 (m, 4H), 4.08 - 4.02 (m, 4H), 3.80 - 3.31 (m, 27H), 1.47 - 1.28 (m, 24H). 13C NMR (175 MHz, MeOD-d$_4$): δ = 110.4, 82.3 - 82.0, 79.9 - 79.8, 76.1 - 76.0, 73.4, 72.5 - 72.1, 71.7, 71.1, 70.6, 67.4 - 67.5, 62.4 - 61.9, 38.1, 27.1, 25.7.

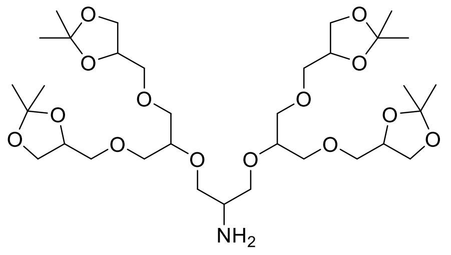

[pG2]-NH$_2$ (aa)

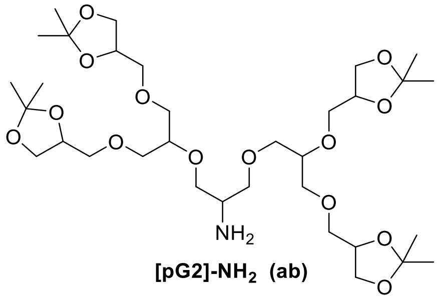

[pG2]-NH$_2$ (ab)

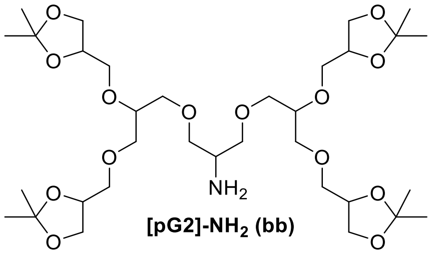

[pG2]-NH$_2$ (bb)

[pG2]-NH$_2$ (aa:ab:bb, 4:4:1).[1] The starting material **[pG2]-N$_3$** (1.30 g, 1.80 mmol, **aa**:**ab**:**bb**, 3:4:1) was dissolved in MeOH (10 mL) and Pd/C (100 mg) was added. The mixture was stirred under hydrogen atmosphere (5 bar) and at room temperature for 16 hours. Subsequently, the mixture was passed through a syringe filter (0.2 µm, RC) and the solvent was removed under reduced pressure to obtain [pG2]-NH$_2$ (1.00 g, 1.43 mmol, **aa**:**ab**:**bb**, 4:4:1, 80%). 1H NMR (700 MHz, MeOD-d$_4$): δ = 4.65 (s, 2H), 4.27 - 4.21 (m, 4H), 4.06 - 4.04 (m, 4H), 3.89 - 3.77 (m, 27H), 1.46 - 1.25 (m, 24H). 13C NMR (175 MHz, MeOD-d$_4$): δ = 110.3, 82.2, 81.9, 79.9 - 79.6, 76.1 - 76.0, 73.8 - 73.7, 73.3, 72.7 - 72.1, 71.6 - 71.5, 70.5, 67.6 - 67.5, 52.2 - 51.6, 27.1, 25.7.

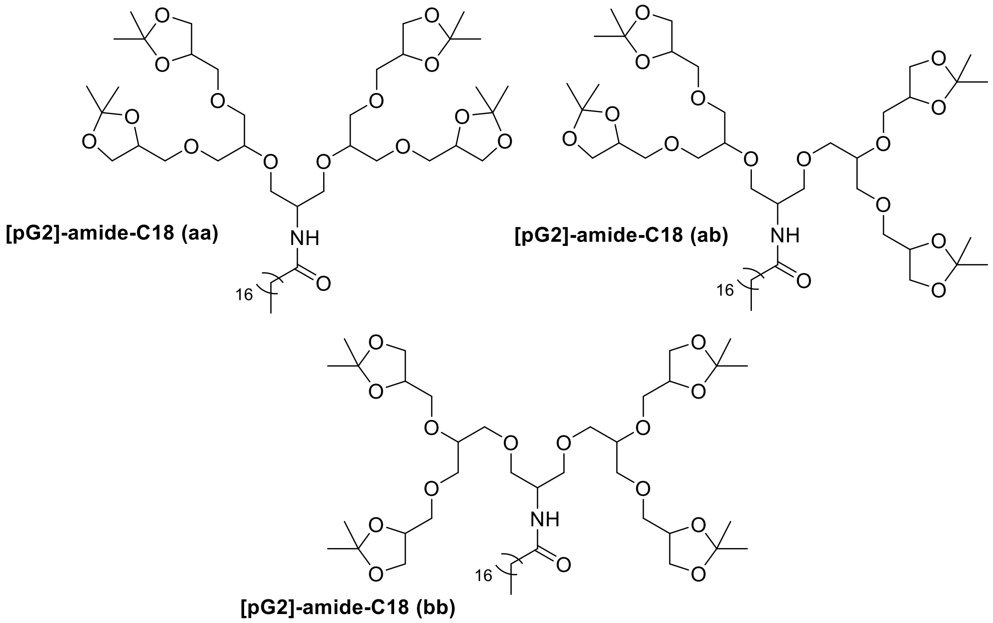

[pG2]-amide-C18 (aa:ab:bb). The starting material **[pG2]-NH$_2$** (1.90 g, 2.87 mmol, **aa**:**ab**:**bb**, 4:4:1) and triethylamine (1.48 mL, 11.5 mmol) were dissolved in DCM (10 mL). The mixture was cooled with an ice bath and stearoyl chloride (1.07 g, 3.55 mmol) was added. The mixture was stirred and allowed to warm up to room temperature over a time period of 16 hours. The mixture was directly purified by column chromatography (SiO_2, DCM/EtOAc, 4:1 → 4:1 + 4% MeOH), which led to the obtainment of **[pG2]-amide-C18** (1.82 g, 1.90 mmol, **aa**:**ab**:**bb**, 66%). 1H NMR (400 MHz, MeOD-d$_4$): δ = 4.29 - 4.20 (m, 4H), 4.08 - 4.01 (m, 4H), 3.79 - 3.46 (m, 27H), 2.26 - 2.16 (m, 2H), 1.65 - 1.55 (m, 2H), 1.42 - 1.24 (m, 52H), 0.95 - 0.87 (m, 3H). 13C NMR (101 MHz, MeOD-d$_4$): δ = 175.7, 110.4 - 110.2, 79.8 - 79.6, 76.1 - 76.0, 73.4 - 73.3, 72.5 - 72.0, 71.2 - 71.0, 70.0 - 69.8, 67.7 - 67.5, 37.1, 33.0, 30.8 - 30.3, 27.2 - 27.0, 25.7, 23.7. MS (ESI): *m/z* = 984.6684 $C_{51}H_{95}N_1O_{15}Na_1^+$ (calculated = 984.6594).

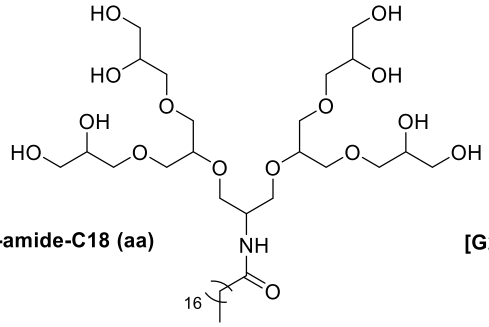

[G2]-amide-C18 (aa)

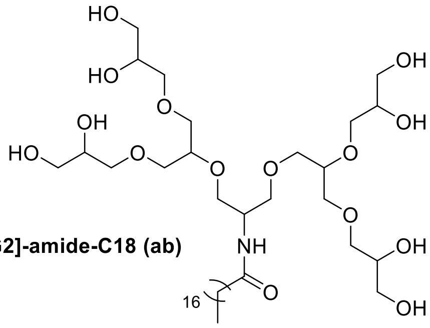

[G2]-amide-C18 (ab)

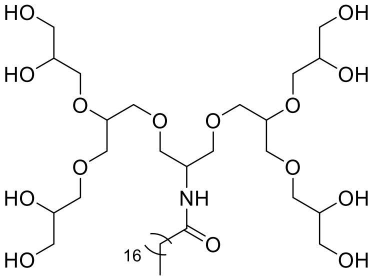

[G2]-amide-C18 (bb)

[G2]-amide-C18 (aa:ab:bb, 4:4:1). The starting material **[pG2]-amide-C18** (1.90 g, 2.87 mmol, **aa**:**ab**:**bb**) was dissolved in MeOH (500 mL) and HCl (37%, 100 µL) was added. The mixture was stirred for 16 hours at room temperature. The solvent was removed under reduced pressure and the procedure was repeated. The raw material was then dissolved in a mixture of water and MeOH (1/1, *v/v*, 25 mL) and passed through a syringe filter (RC, 0.2 µm). Subsequent purification by means of reversed-phase HPLC (water/MeOH, 1/4) led to the obtainment of **[G2]-amide-C18** (1.28 g, 1.60 mmol, **aa**:**ab**:**bb**, 4:4:1, 56%). 1H NMR (700 MHz, MeOD-d_4): δ = 4.19 - 4.08 (m, 1H), 3.79 - 3.74 (m, 4H), 3.71 - 3.65 (m, 5H), 3.63 - 3.44 (m, 25H), 2.25 - 2.17 (m, 2H), 1.66 - 1.55 (m, 2H), 1.41 - 1.21 (m, 28H), 0.94 - 0.86 (m, 3H). 13C NMR (175 MHz, MeOD-d_4): δ = 176.0, 79.6 - 79.5, 73.8, 72.8, 72.2 - 72.0, 71.0, 69.8, 64.3, 50.8 - 50.0, 37.0, 32.9, 30.7 - 30.0, 27.0, 23.6, 14.5. MS (ESI): *m/z* = 824.5364 $C_{39}H_{79}N_1O_{15}Na_1^+$ (calculated = 824.5342).

3.3.8 [G2] OGD Regioisomer Mixture 7

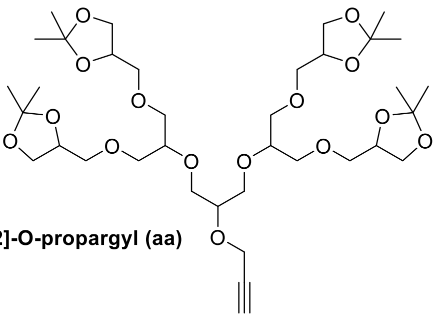

[pG2]-O-propargyl (aa)

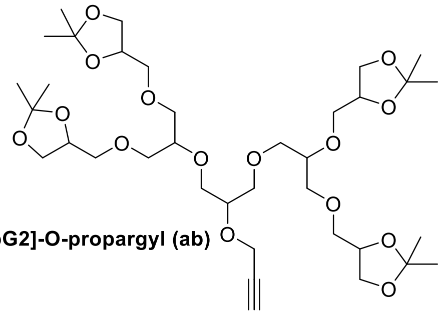

[pG2]-O-propargyl (ab)

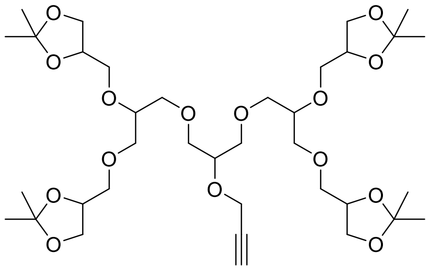

[pG2]-O-propargyl (bb)

[pG2]-O-propargyl (aa:ab:bb, 4:4:1).[1] The starting material [pG2]-OH (2.00 g, 2.80 mmol, **aa**:**ab**:**bb**, 4:4:1) was dried under reduced pressure (~ 10^{-2} mbar) and dissolved in DMF (70 mL). The flask was cooled with an ice bath and NaH (60w%, 0.35 g, 14.4 mmol) was added in small portions. The ice bath was removed and the mixture was heated up to 60 °C for 30 minutes. The temperature was reduced to 40 °C before 15-crown-5 (catalytic amounts) and propargyl bromide (80w%, 1.56 mL, 14.0 mmol) were added. The mixture was stirred at 40 °C for 12 hours before a saturated aqueous solution of NH_4Cl (50 mL) was slowly added. The solvent was removed under reduced pressure and the residue was mixed with water (200 mL), EtOAc (150 mL), and Brine (100 mL). The aqueous layer was extracted with EtOAc (3 x 150 mL), the organic layer was dried over Na_2SO_4, and the solvent was removed under reduced pressure. Column chromatography (SiO_2, *n*-pentane/EtOAc, 3/1 → 3/1 + 2% MeOH) gave **[pG2]-O-propargyl** (1.60 g, 2.17 mmol, **aa**:**ab**:**bb**, 4:4:1, 78%). 1H NMR (400 MHz, MeOD-d_4): δ = 4.35 - 4.31 (m, 2H), 4.28 - 4.21 (m, 4H), 4.08 - 4.02 (m, 4H), 3.79 - 3.50 (m, 27H), 2.89 - 2.85 (m, 1H), 1.41 - 1.29 (m, 24H). 13C NMR (101 MHz, MeOD-d_4): δ = 110.3, 81.3 - 81.2, 79.8 - 79.7, 78.4 - 77.7, 76.7 - 75.7, 73.3, 72.4 - 72.3, 71.1, 67.7 - 67.5, 58.2, 27.2 - 27.1, 25.7. MS (ESI): *m/z* = 757.4073 $C_{36}H_{62}O_{15}Na_1^+$ (calculated = 757.3981).

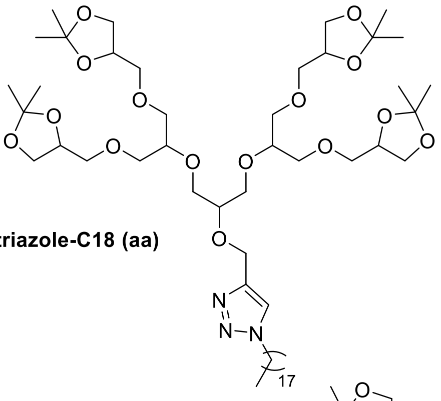

[pG2]-triazole-C18 (aa)

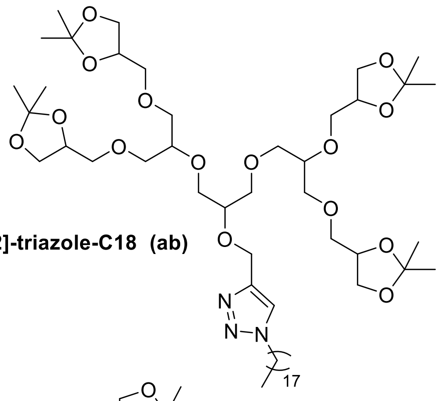

[pG2]-triazole-C18 (ab)

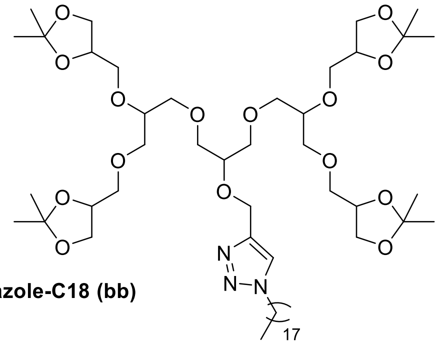

[pG2]-triazole-C18 (bb)

[pG2]-triazole-C18 (aa:ab:bb). The starting material **[pG2]-O-propargyl** (1.60 g, 2.17 mmol, **aa**:**ab**:**bb**, 4:4:1) and 1-azidooctadecane (0.70 g, 2.30 mmol) were dissolved in a mixture of THF (5 mL) and water (3 mL). Diisopropylamine (37.0 µL, 0.21 mmol), sodium ascorbate (171 mg, 0.86 mmol, dissolved in 1 mL water), and Cu(II)SO4*5H_2O (54.1 mg, 0.21 mmol, dissolved in 1 mL water) were added. The mixture was stirred at room temperature for 16 hours and was then diluted with water (60 mL). A saturated aqueous solution of EDTA (1 mL) and Brine (20 mL) were added. The aqueous layer was extracted with EtOAc (3 x 50 mL). The organic layer was dried over Na_2SO_4 and the solvent was removed under reduced pressure. Column chromatography (SiO_2, DCM/EtOAc, 4/1 + 2% MeOH) gave the desired product (1.88 g, 1.82 mmol, **aa**:**ab**:**bb**, 84%). 1H NMR (400 MHz, MeOD-d_4): δ = 7.98 (m, 1H), 4.80 - 4.76 (m, 2H), 4.43 - 4.36 (m, 2H), 4.28 - 4.19 (m, 2H), 4.06 - 4.00 (m, 4H), 3.77 - 3.47 (m, 29H), 1.96 - 1.86 (m, 2H), 1.41 - 1.26 (m, 54H), 0.93 - 0.87 (m, 3H). 13C NMR (101 MHz, MeOD-d_4): δ = 146.4, 124.8, 110.3, 79.8, 79.2, 78.9, 76.1 - 76.0, 73.4, 72.5 - 72.3, 71.2, 67.7 - 67.5, 64.3, 61.4, 51.3, 33.0, 31.3, 30.8 - 30.4, 30.1, 27.5, 27.2 - 27.1, 25.7, 23.7, 14.5. MS (ESI): *m/z* = 1052.6990 $C_{54}H_{99}N_3O_{15}Na_1^+$ (calculated = 1052.6968).

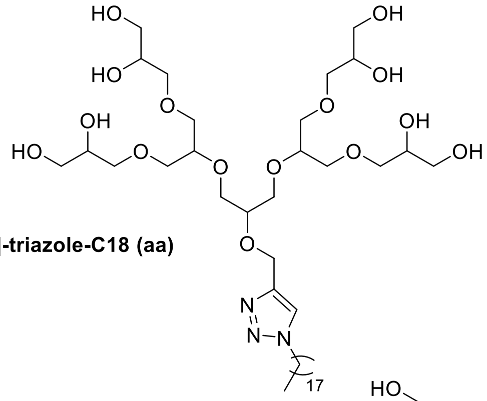

[G2]-triazole-C18 (aa)

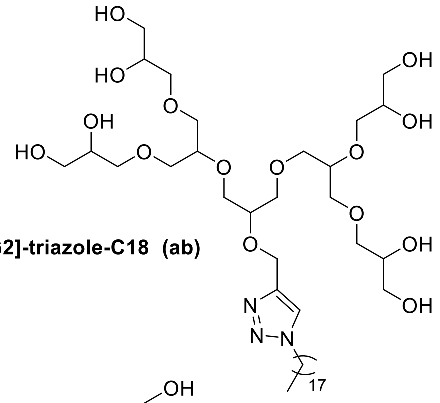

[G2]-triazole-C18 (ab)

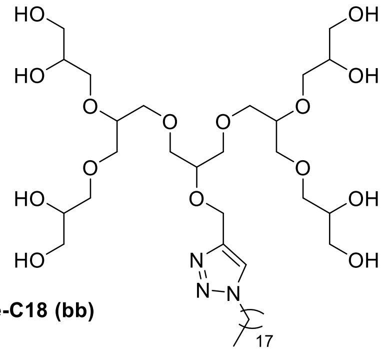

[G2]-triazole-C18 (bb)

[G2]-triazole-C18 (aa:ab:bb, 1:2:1). The starting material **[pG2]-triazole-C18** (1.88 g, 1.82 mmol, **aa**:**ab**:**bb**) was dissolved in MeOH (500 mL) and treated with HCl (37%, 100 μL). The mixture was stirred for 16 hours at room temperature, the solvent was removed under reduced pressure, and the procedure was repeated. The crude product was then dissolved in a mixture of water and MeOH (1/1, *v/v*, 40 mL), passed through a syringe filter (RC, 0.2 μm), and purified by means of reversed-phase HPLC (water/MeOH, 1/9) to obtain **[G2]-triazole-C18** (1.26 g, 1.44 mmol, **aa**:**ab**:**bb**, 1:2:1, 80%). 1H NMR (400MHz, MeOD-d_4): δ = 8.03 (m, 1H), 4.82 - 4.75 (m, 2H), 4.44 - 4.36 (m, 2H), 3.83 - 3.44 (m, 35H), 1.95 - 1.86 (m, 2H), 1.39 - 1.21 (m, 30H), 0.93 - 0.86 (m, 3H). 13C NMR (101 MHz, MeOD-d_4): δ = 146.1, 125.0, 79.7, 79.1, 78.8, 73.8, 72.8, 72.3 - 72.0, 71.0, 64.3 - 64.1, 51.3, 33.0, 31.3, 30.7 - 30.4, 30.0, 27.4, 23.6, 14.5. MS (ESI): *m/z* = 892.5715 $C_{42}H_{83}N_3O_{15}Na_1^+$ (calculated = 892.5716).

3.4 Collision Cross Section Calculation

Conformational search and optimization of the head groups of **4a** and **4b** were achieved using the MM2 force field as implemented in ChemBio3D v14.0 (PerkinElmer). The pdb data of the head groups are listed below. Theoretical collision cross section (CCS) values were calculated from the pdb data using the projection approximation algorithm.[6]

pdb data of the head group 4a:

```
REMARK   This PDB file was created by CS Chem3D.
HETATM    1  O         1      -1.038  -1.297   0.357                    O
HETATM    2  C         1      -0.839  -2.687   0.310                    C
HETATM    3  C         1       0.068  -3.047  -0.872                    C
HETATM    4  C         1       1.458  -2.402  -0.821                    C
HETATM    5  O         1       1.345  -1.037  -1.132                    O
HETATM    6  C         1       2.552  -0.327  -0.998                    C
HETATM    7  C         1       2.270   1.144  -1.267                    C
HETATM    8  C         1      -1.886   0.625   1.460                    C
HETATM    9  C         1      -1.914  -0.894   1.382                    C
HETATM   10  C         1       1.187   1.695  -0.360                    C
HETATM   11  C         1      -2.454   1.308   0.229                    C
HETATM   12  O         1      -2.635   1.061   2.570                    O
HETATM   13  O         1      -2.178   2.691   0.327                    O
HETATM   14  O         1       3.433   1.905  -1.044                    O
HETATM   15  O         1       1.051   3.082  -0.586                    O
HETATM   16  H         1      -0.365  -3.023   1.262                    H
HETATM   17  H         1      -1.825  -3.194   0.197                    H
HETATM   18  H         1       0.194  -4.158  -0.884                    H
HETATM   19  H         1      -0.435  -2.773  -1.830                    H
HETATM   20  H         1       2.131  -2.880  -1.571                    H
HETATM   21  H         1       1.896  -2.530   0.195                    H
HETATM   22  H         1       2.941  -0.450   0.039                    H
HETATM   23  H         1       3.299  -0.723  -1.724                    H
HETATM   24  H         1       1.982   1.294  -2.334                    H
HETATM   25  H         1      -0.839   0.963   1.641                    H
HETATM   26  H         1      -1.572  -1.322   2.352                    H
HETATM   27  H         1      -2.943  -1.256   1.158                    H
HETATM   28  H         1       0.207   1.231  -0.591                    H
HETATM   29  H         1       1.429   1.549   0.716                    H
HETATM   30  H         1      -2.001   0.942  -0.717                    H
HETATM   31  H         1      -3.557   1.183   0.162                    H
HETATM   32  H         1      -2.597   2.021   2.591                    H
HETATM   33  H         1      -2.633   3.159  -0.376                    H
HETATM   34  H         1       3.176   2.832  -1.066                    H
HETATM   35  H         1       0.289   3.403  -0.097                    H
CONECT    1    2    9
CONECT    2    1    3   16   17
CONECT    3    2    4   18   19
CONECT    4    3    5   20   21
CONECT    5    4    6
CONECT    6    5    7   22   23
CONECT    7    6   10   14   24
CONECT    8    9   11   12   25
CONECT    9    8    1   26   27
CONECT   10    7   15   28   29
CONECT   11    8   13   30   31
```

```
CONECT   12    8   32
CONECT   13   11   33
CONECT   14    7   34
CONECT   15   10   35
CONECT   16    2
CONECT   17    2
CONECT   18    3
CONECT   19    3
CONECT   20    4
CONECT   21    4
CONECT   22    6
CONECT   23    6
CONECT   24    7
CONECT   25    8
CONECT   26    9
CONECT   27    9
CONECT   28   10
CONECT   29   10
CONECT   30   11
CONECT   31   11
CONECT   32   12
CONECT   33   13
CONECT   34   14
CONECT   35   15
END
```

pdb data of the head group 4b:

```
REMARK   This PDB file was created by CS Chem3D.
HETATM    1  C          1      -1.820   0.669  -0.183                C
HETATM    2  C          1      -1.600  -0.658  -0.895                C
HETATM    3  O          1      -0.646  -1.409  -0.185                O
HETATM    4  O          1      -0.626   1.413  -0.216                O
HETATM    5  C          1       0.593   3.374   0.366                C
HETATM    6  C          1      -0.755   2.672   0.396                C
HETATM    7  C          1      -0.408  -2.668  -0.763                C
HETATM    8  C          1       0.675  -3.378   0.033                C
HETATM    9  C          1       1.124   3.600  -1.036                C
HETATM   10  C          1       0.305  -3.605   1.486                C
HETATM   11  O          1       0.472   4.638   0.975                O
HETATM   12  O          1       2.270   4.421  -0.937                O
HETATM   13  O          1       0.917  -4.643  -0.537                O
HETATM   14  O          1       1.296  -4.433   2.061                O
HETATM   15  H          1      -2.624   1.242  -0.701                H
HETATM   16  H          1      -2.121   0.490   0.875                H
HETATM   17  H          1      -2.560  -1.225  -0.932                H
HETATM   18  H          1      -1.240  -0.479  -1.934                H
HETATM   19  H          1       1.340   2.799   0.963                H
HETATM   20  H          1      -1.085   2.539   1.453                H
HETATM   21  H          1      -1.513   3.276  -0.153                H
HETATM   22  H          1      -1.348  -3.266  -0.748                H
HETATM   23  H          1      -0.072  -2.535  -1.817                H
HETATM   24  H          1       1.633  -2.809  -0.027                H
HETATM   25  H          1       1.427   2.650  -1.529                H
HETATM   26  H          1       0.387   4.132  -1.678                H
HETATM   27  H          1       0.277  -2.656   2.066                H
HETATM   28  H          1      -0.670  -4.131   1.588                H
HETATM   29  H          1       1.295   5.112   0.816                H
```

```
HETATM   30  H          1       2.655   4.553  -1.806                     H
HETATM   31  H          1       1.496  -5.122   0.063                     H
HETATM   32  H          1       1.112  -4.566   2.993                     H
CONECT    1    2    4   15   16
CONECT    2    1    3   17   18
CONECT    3    2    7
CONECT    4    1    6
CONECT    5    6    9   11   19
CONECT    6    5    4   20   21
CONECT    7    3    8   22   23
CONECT    8    7   10   13   24
CONECT    9    5   12   25   26
CONECT   10    8   14   27   28
CONECT   11    5   29
CONECT   12    9   30
CONECT   13    8   31
CONECT   14   10   32
CONECT   15    1
CONECT   16    1
CONECT   17    2
CONECT   18    2
CONECT   19    5
CONECT   20    6
CONECT   21    6
CONECT   22    7
CONECT   23    7
CONECT   24    8
CONECT   25    9
CONECT   26    9
CONECT   27   10
CONECT   28   10
CONECT   29   11
CONECT   30   12
CONECT   31   13
CONECT   32   14
END
```

3.5 Pendant Drop Method

Pendant drop experiments were performed as described before.[5, 7] Briefly, a dilution series was prepared and the samples were investigated using a contact angle tensiometer OCA 20 (DataPhysics Instruments GmbH, Germany). To reduce the impact of evaporation effects, a wet filter paper was placed in a watch glass on the bottom of the measurement chamber. The dispensed droplet (volume = 18 – 20 µL) was equilibrated between 30 and 90 minutes at room temperature (22 - 23 °C) before a constant interfacial surface tension (IFT) was obtained. IFT values were determined for every concentration from three independent droplets. The IFT values were averaged and plotted with their standard deviation against the logarithm of the concentration (see Figure 3, manuscript). The error bars are smaller than the size of the plotted dots.

3.6 Estimation of logP Values

The logP values were estimated from the molecular structures of individual regioisomers using ChemDraw Professional (v19.1.1.21).

3.7. Critical Aggregation Concentration

Critical aggregation concentration (*cac*) values were determined by dynamic light scattering (DLS) using previously published procedures:[1, 4, 8] Serial dilutions with OGD concentrations between 10^{-8} and 10^{-2} M were prepared in MilliQ water. The samples were filtered (RC, 0.2 µm) and equilibrated for 16 hours at room temperature prior to their analysis. The samples were transferred into a quartz cuvette (Quartz Suprasil, width × length: 2 mm × 10 mm) and analysed with a Zetasizer Nano-ZS ZEN3600 (Malvern, UK). The instrumental parameters were as follows: material (polystyrene latex), dispersant (water), sample viscosity parameters (use dispersant viscosity as sample viscosity), temperature (22.5 °C), equilibration time (120 s), cell type (quartz cuvettes), measurement angle (173° backscatter), measurement duration (manual), number of runs (11), run duration (10 s), number of measurements (3), delay between the measurements (0 s), data processing (general purpose, normal resolution). The derived count rate values obtained from three measurements per concentration were averaged. The unit of the derived count rate is kilo counts per second (kcps). The logarithm of the derived count rate was plotted against the logarithm of the concentration. The double logarithmic plots showed two characteristic regions: (1) a flat region with low count rates at lower concentrations and (2) a linear growth of the count rate at higher concentrations. Both regions were fitted to linear functions and the intersection was taken as the *cac* value. An image visualizing the fitting procedure is provided in Figure S9 (Supporting information) of another article – see Ref.[4].

3.8 Membrane Protein Purification

The relative protein quantities obtained upon IMAC purification discussed throughout this paper have been obtained from a previously published paper (Table S4).[1] Briefly, the membrane proteins were expressed in *Escherichia Coli* (*E. coli*) and purified from bacterial membranes using *n*-dodecyl-ß-D-maltoside (DDM) and [G1] OGD batches **3** (= **3a** + **3b**), **3a**, and **3b**. Protein solutions obtained upon IMAC were concentrated to equal volumes and relative protein quantities were determined by UV/VIS spectroscopy. Absorbance values (A_{485} for AqpZ-GFP, A_{280} for AmtB-MBP) were normalized to those obtained from DDM, averaged (n = 3), and plotted with standard deviation (±SD) against the detergent abbreviation (Figure 3, Figure S8). The data are summarized in Table S4. For further information about the experimental procedure see Urner *et al.*[1]

3.9 Monitoring the Activity of Outer Membrane Protease T

The outer membrane protease was refolded as described before.[1] The activity assay was also performed in analogy to a previously published procedure:[1] The activity of OmpT was assessed by monitoring the time-dependent cleavage of a self-quenching fluorescent peptide Abz-ARRAY-Tyr(NO_2)-NH_2 (Biomatik, custom synthesis) in which “Abz” abbreviates *o*-aminobenzoyl and “Tyr(NO_2)” abbreviates 3-nitrotyrosine[9]. The following components were mixed in chambers of a 96 well plate (Greiner 96F-Bottom): assay buffer (233.5 µL of 10 mM Bis-Tris, 5 mM EDTA, pH = 6.5), OmpT (10 µL of a 10 µM OmpT solution in 100 mM (NH_4)HCO_3, pH = 8), LPS (10 µL of a 5 mg/mL solution in H_2O), and Abz-ARRAY-Tyr(NO_2)-NH_2 (9 µL of a 980 µM aqueous solution). Assay buffers and protein solutions contained detergent (1xcac). The LPS solution was added last and all ingredients were mixed before analysis resulting in a dead time of about 30 s. Time-dependent cleavage of the peptide was monitored with a CLARIOstar microplate reader (BMG Labtech). The following experimental parameters were used: bottom optic, focal height (4.1 mm), excitation wavelength (325 nm), emission wavelength (430 nm), number of cycles (70), cycle duration (10 s), temperature (26 °C), No. of flashed per well (20), gain (1000), and settling time (0.5 s). Data were acquired with CLARIOstar® V5.4 and analysed with MARS V3.3 and Origin V9.1.

4. Literature

[1] L. H. Urner, I. Liko, H.-Y. Yen, K. K. Hoi, J. R. Bolla, J. Gault, F. G. Almeida, M.-P. Schweder, D. Shutin, S. Ehrmann, R. Haag, C. V. Robinson, K. Pagel, *Nat. Commun.* **2020**, *11*, 1 - 10.

[2] a) M. Wyszogrodzka, R. Haag, *Chem. Eur. J.* **2008**, *14*, 9202 - 9214; b) M. Wyszogrodzka, K. Möws, S. Kamlage, J. Wodzinska, N. Plietker, R. Haag, *Eur. J. Org. Chem.* **2008**, 53-63; c) F. Paulus, R. Schulze, D. Steinhilber, M. Zieringer, I. Steinke, P. Welker, K. Licha, S. Wedepohl, J. Dernedde, R. Haag, *Macromol. Biosci.* **2014**, *14*, 643-654.

[3] L. H. Urner, S. Ehrmann, R. Haag, K. Pagel, H.-Y. Yen, I. Liko, C. V. Robinson, **2020**, WO 2020/049294A049291.

[4] L. H. Urner, M. Schulze, Y. B. Maier, W. Hoffmann, S. Warnke, I. Liko, K. Folmert, C. Manz, C. V. Robinson, R. Haag, K. Pagel, *Chem. Sci.* **2020**, *11*, 3538-3546.

[5] L. H. Urner, B. Schade, M. Schulze, K. Folmert, R. Haag, K. Pagel, *ChemPhysChem.* **2019**, *20*, 1690-1697.

[6] G. von Helden, M. T. Hsu, N. Gotts, M. T. Bowers, *J. Phys. Chem.* **1993**, *97*, 8182-8192.

[7] L. H. Urner, B. N. S. Thota, O. Nachtigall, S. Warnke, G. von Helden, R. Haag, K. Pagel, *Chem. Commun.* **2015**, *51*, 8801-8804.

[8] Y. Skhiri, P. Gruner, B. Semin, Q. Brosseau, D. Pekin, L. Mazutis, v. Goust, F. Kleinschmidt, A. El Harrak, J. B. Hutchison, E. Mayot, J.-F. Bartolo, A. D. Griffiths, V. Taly, J.-C. Baret, *Soft Matter.* **2012**, *8*, 10618-10627.

[9] R. A. Kramer, D. Zandwijken, M. R. Egmond, N. Dekker, *Eur. J. Biochem.* **2000**, *267*, 885-893.
